# Constitutive cyclic GMP accumulation in *Arabidopsis thaliana* compromises systemic acquired resistance induced by an avirulent pathogen by modulating local signals

**DOI:** 10.1038/srep36423

**Published:** 2016-11-04

**Authors:** Jamshaid Hussain, Jian Chen, Vittoria Locato, Wilma Sabetta, Smrutisanjita Behera, Sara Cimini, Francesca Griggio, Silvia Martínez-Jaime, Alexander Graf, Mabrouk Bouneb, Raman Pachaiappan, Paola Fincato, Emanuela Blanco, Alex Costa, Laura De Gara, Diana Bellin, Maria Concetta de Pinto, Elodie Vandelle

**Affiliations:** 1 Department of Biotechnology, University of Verona, Verona, Italy; 2 Unit of Food Science and Nutrition, Department of Medicine, University Campus Bio-Medico of Rome, Rome, Italy; 3 Institute of Biosciences and Bioresources – National Research Council, Bari, Italy; 4 Department of Biosciences, University of Milan, Milan, Italy; 5 Max Planck Institute of Molecular Plant Physiology, Potsdam-Golm, Germany; 6 Department of Biology, University of Bari “Aldo Moro”, Bari, Italy

## Abstract

The infection of *Arabidopsis thaliana* plants with avirulent pathogens causes the accumulation of cGMP with a biphasic profile downstream of nitric oxide signalling. However, plant enzymes that modulate cGMP levels have yet to be identified, so we generated transgenic *A. thaliana* plants expressing the rat soluble guanylate cyclase (GC) to increase genetically the level of cGMP and to study the function of cGMP in plant defence responses. Once confirmed that cGMP levels were higher in the GC transgenic lines than in wild-type controls, the GC transgenic plants were then challenged with bacterial pathogens and their defence responses were characterized. Although local resistance was similar in the GC transgenic and wild-type lines, differences in the redox state suggested potential cross-talk between cGMP and the glutathione redox system. Furthermore, large-scale transcriptomic and proteomic analysis highlighted the significant modulation of both gene expression and protein abundance at the infection site, inhibiting the establishment of systemic acquired resistance. Our data indicate that cGMP plays a key role in local responses controlling the induction of systemic acquired resistance in plants challenged with avirulent pathogens.

Plants respond to the presence of microorganisms by deploying efficient defence mechanisms leading to incompatible plant–pathogen interactions. These mechanisms include a basal defence response following the recognition of common microbial features, known as pathogen-associated molecular patterns or microbe-associated molecular patterns. If this is insufficient, plants have evolved a second layer of strong and potent defences known as the hypersensitive response (HR), which is triggered by the specific recognition of pathogen effectors (Avr) by resistance (R) proteins located on the plasma membrane or in the cytosol.

Among the first events triggered by the HR is the simultaneous production of reactive oxygen species (ROS)^1^ and nitric oxide (NO)^2^,^3^,^4^. These act in a coordinated manner, according to the so-called ‘balance model’, to induce hypersensitive programmed cell death at the infection site to restrict pathogen growth^5^. As well as inducing cell death, NO regulates the expression of a battery of defence genes allowing the establishment of local resistance, and also systemic acquired resistance (SAR)^6^. The latter is a broad and long-lasting systemic response that protects uninfected tissues against subsequent infections by diverse pathogens, and this requires the careful balancing of phytohormone cross-talk, which strongly influences the outcome of plant immunity^7^,^8^. The induction of SAR requires the generation of a local signal in infected cells, followed by the transduction of this signal systemically through the vasculature, generally the phloem^9^. During this process, NO and ROS act in parallel with salicylic acid (SA) signalling upstream of AzA/G3P and fatty acids for the induction of SAR^6^.

NO signalling can be mediated by the direct interaction between NO and target molecules to introduce post-translational modifications^10^,^11^,^12^. Alternatively, NO can induce second messengers such as Ca^2+^ or cyclic guanosine 3′,5′-monophosphate (cGMP) to regulate defence gene expression^13^. The presence of cGMP in plants has been reported in previous studies^14^,^15^,^16^,^17^,^18^, and it can induce defence gene expression in tobacco, indicating an important role in plant defence responses^3^. More recently, cGMP has been shown to accumulate especially during incompatible interactions induced by avirulent bacterial pathogens (*Pseudomonas syringae* DC3000 carrying the *AvrB* gene) that typically elicit the HR in *Arabidopsis thaliana*^17^. The synthesis of cGMP may be required for NO-induced cell death, although the analogue 8-Br-cGMP alone cannot induce cell death or potentiate cell death triggered by a NO donor^19^. Taken together, these results suggest that cGMP could play a key role in plant defences, particularly the mediation of NO-dependent responses.

Despite the pathophysiological data described above, there is no genetic evidence as yet to support the role of cGMP in plant defence responses. Indeed, despite evidence for the presence of cGMP in plants, the guanylate cyclase (GC) and phosphodiesterase (PDE), which catalyse its synthesis and degradation in other eukaryotes, have yet to be identified in plants^20^. Eukaryotic GCs are highly conserved and contain a specific consensus motif which has led to the identification of several candidate plant enzymes by homology searching, namely AtGC1, AtBRI1, AtWAKL10, AtPSKR1, AtPepR1, AtNOGC1 and more recently the plant natriuretic peptide receptor^21^,^22^,^23^,^24^,^25^,^26^,^27^. However, only AtNOGC1 binds NO, inducing the NO-dependent production of cGMP *in vitro*^26^. The GC activity of all these proteins is also extremely low compared to animal GCs, raising doubts concerning their physiological relevance for cGMP biosynthesis in plants^28^. A cGMP-specific PDE activity has been detected in *A. thaliana* seedlings^29^ but the corresponding gene(s) have not been isolated.

To study the role of cGMP in NO-dependent plant defence responses in the absence of relevant endogenous enzymes, we developed an alternative strategy involving the creation of transgenic *A. thaliana* plants expressing mammalian soluble GC (GC). We characterized the transgenic plants, which produced higher levels of cGMP than wild-type controls, to investigate their defence responses when challenged with bacterial pathogens.

## Results

### Avirulent *PstAvrB* induces a specific NO-dependent increase in cGMP levels

**Figure 1:**
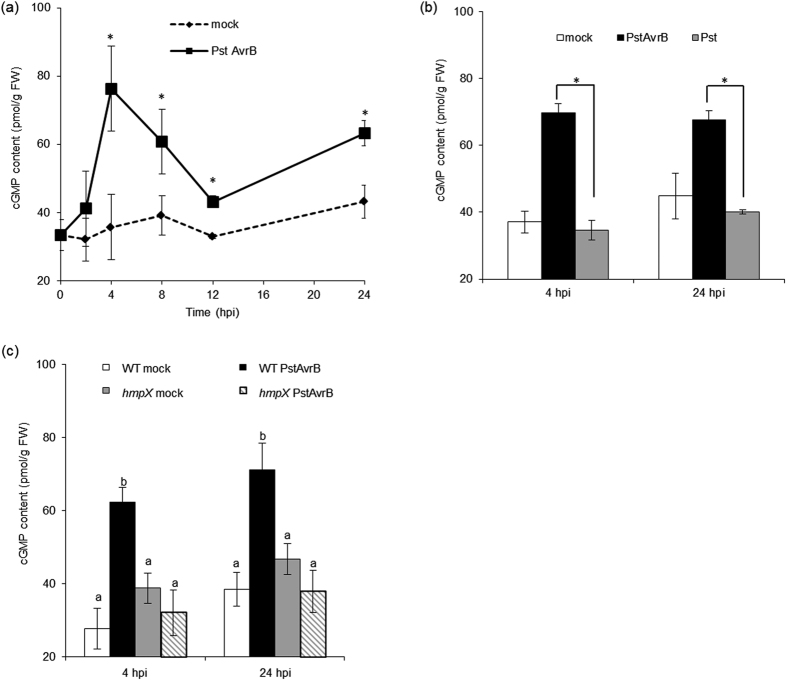
Variation in cGMP levels in *A. thaliana* plants over time in response to *PstAvrB*. Measurement of the cGMP content in (**a**) *A. thaliana* Col-0 leaves infiltrated with *PstAvrB* (10^7^ cfu/mL) and collected at different time points during the course of infection, (**b**) *A. thaliana* Col-0 leaves infiltrated with *PstAvrB* (10^7^ cfu/mL) or *Pst* (10^7^ cfu/mL) at 4 and 24 hpi, (**c**) *A. thaliana* Col-0 and *hmpX* transgenic plants infiltrated with *PstAvrB* (10^7^ cfu/mL) at 4 and 24 hpi. In each experiment, mock-infiltrated plants were used as controls. Values are means ± SEM of at least three biological replicates, each including three technical replicates. FW: fresh weight. Asterisks or different letters indicate a statistical difference (p < 0.05) according to Student’s *t*-test (**a,b**) or ANOVA test (**c**).

To monitor the concentration of cGMP in *A. thaliana* plants we developed a new technique based on Perkin Elmer AlphaScreen technology. The method optimized in this work for plant samples was able to detect cGMP increase in *A. thaliana* leaves infiltrated with the cGMP-specific phosphodiesterase inhibitor sildenafil 1 mg/mL^30^, reaching around 70 pmol/g FW 4 hours post-infiltration (hpi) (Supplementary Fig. S1a). Then during the course of infection with an avirulent strain of *Pseudomonas syringae* pv. *tomato* carrying the avirulent gene *AvrB* (*PstAvrB*), we found that cGMP accumulated in a biphasic manner, with an initial peak 4  hpi followed by a decrease to basal levels and a subsequent increase at around 24 hpi (Fig. 1a). The accumulation of cGMP at each time point appeared strictly related to the HR because there was no increase following infection with virulent *Pst* (Fig. 1b). It is worth mentioning that the levels of cGMP detected in this work are around 100 fold higher than the ones reported previously by Meier and colleagues^17^. Such difference could be attributable to the methods used for monitoring cGMP in both works. However, the concentrations detected here are of the same order of magnitude than cGMP concentrations reported so far in other plant species, such as tobacco leaves (10 pmol/g FW; ref. 3), spruce needles (20 pmol/g FW; ref. 31), mung bean explants (40 pmol/g FW, ref. 18).

In addition, the increase in cGMP levels induced by *PstAvrB* was shown to be NO-dependent because cGMP did not accumulate in transgenic *A. thaliana hmpX* plants expressing the bacterial NO dioxygenase, which detoxifies NO^32^ (Fig. 1c). Similarly there was no increase following the co-infiltration of wild-type *A. thaliana* plant leaves with avirulent *PstAvrB* together with the NO scavenger cPTIO (Supplementary Fig. S1b). Together these data confirmed that cGMP accumulates specifically during the HR downstream of NO signal, potentially to mediate defence responses.

### High constitutive cGMP content does not affect local resistance to bacterial infection or hypersensitive cell death but compromises defence gene expression in infected leaves

Homozygous transgenic *A. thaliana* lines expressing the alpha and beta subunits of rat soluble GC were produced by the floral dip method, and lines 6, 26 and 27 showed the expression of both enzyme subunits (Supplementary Fig. S2a). Furthermore expression levels were strongly correlated with cGMP levels that were higher respect to wild-type plants (Supplementary Fig. S2b), thus demonstrating enzyme functionality. However, whereas no significant variations were evident in lines GC6 and GC27 after infection with avirulent *PstAvrB*, a strong decrease of cGMP level was observed in line GC26 following bacterial infection (Supplementary Fig. S3). For that reason, further characterizations were carried on with lines GC6 and GC27.

**Figure 2:**
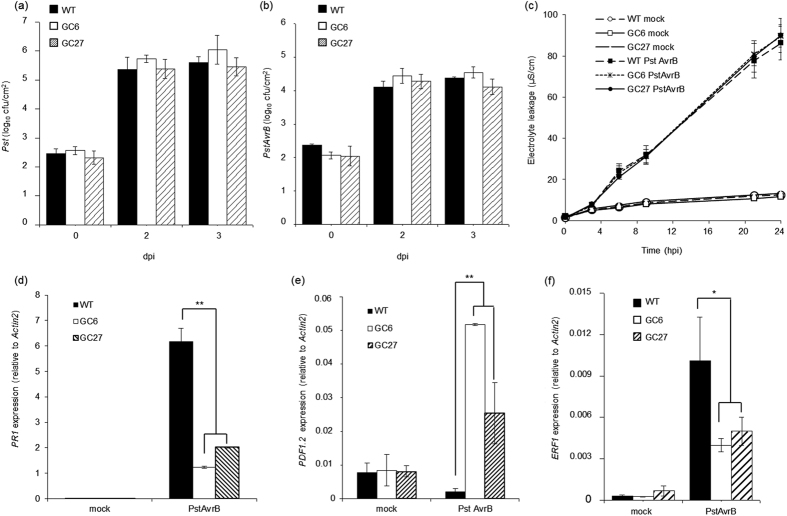
Plants accumulating cGMP show normal local resistance and hypersensitive cell death but dysregulated defence-related gene expression. Plant resistance was assessed by bacterial counting following the infection of *A. thaliana* Col-0, GC6 or GC27 plants with (**a**) virulent *Pst* (5 × 10^5^ cfu/mL) or (**b**) avirulent *PstAvrB* (5 × 10^5^ cfu/mL). (**c**) Hypersensitive cell death was quantified by measuring ion leakage in *A. thaliana* Col-0, GC6 or GC27 plants infected with *PstAvrB* (10^7^ cfu/mL). (**d–f**) *A. thaliana* Col-0, GC6 or GC27 plants were infiltrated with *PstAvrB* (10^7^ cfu/mL) and leaf samples were collected 12 hpi for the analysis of the expression of *PR-1* (**d**), *PDF1.2* (**e**) and *ERF1* (**f**) by real-time RT-PCR using gene-specific primers. The expression level of each gene was normalized to that of *Actin2*. In each experiment, mock-infiltrated plants were used as controls. Values are means ± SEM of at least three biological replicates. Asterisks indicate a statistical difference (*p-value < 0.1; **p-value < 0.05) according to Student’s *t*-test.

Because cGMP potentially acts as a defence signalling molecule, we tested whether the higher level of cGMP in GC lines could improve basal resistance. Wild-type and GC plants were therefore infected with virulent *Pst* (5 × 10^5^ cfu/mL) and resistance was assessed by measuring bacterial growth 2 and 3 days after infection. Figure 2a shows that no difference in bacterial growth was observed between wild-type and both GC lines, indicating that elevated cGMP level alone was not enough to enhance basal resistance. Wild-type, GC6 and GC27 plants were also infected with avirulent *PstAvrB* (5 × 10^5^ cfu/mL) and scored for bacterial growth. Once again, no difference in bacterial counts was observed between the lines (Fig. 2b), indicating that higher cGMP level was not sufficient to enhance specific gene-for-gene resistance.

Based on the proposed role of cGMP in NO-mediated hypersensitive cell death^19^, ion leakage experiments were carried out to determine whether the higher cGMP content in lines GC6 and GC27 enhanced cell death in response to avirulent pathogens. Wild-type and GC plants were infected with *PstAvrB* (10^7^ cfu/mL) and conductivity was measured at different time points after infection to determine the extent of cell death. No difference in ion leakage was observed between the lines, indicating that high cGMP levels do not enhance hypersensitive cell death (Fig. 2c).

To understand whether GC plants were able to induce a full defence response at the molecular level, we analysed the expression of the SA-dependent defence marker gene *PR-1*. Wild-type and GC leaves were infected with avirulent *PstAvrB* and expression analysis was carried out 12 hpi. Surprisingly, the *PstAvrB*-induced upregulation of *PR-1* was significantly muted in both GC6 and GC27 plants compared to wild-type plants (Fig. 2d). Hormone-dependent signalling pathways in plants are differentially regulated in response to distinct types of pathogens, in particular biotrophic and necrotrophic pathogens^7^. We therefore investigated the role of cGMP in the cross-talk between different hormonal signalling pathways by monitoring the expression of *PDF1.2* and *ERF1*, which are related to the jasmonic acid (JA)-dependent and ethylene-dependent pathways, respectively. The genes were analysed 12 h after infection with *PstAvrB* (10^7^ cfu/mL). Interestingly, *PDF1.2* was upregulated in both infected GC lines, whereas *ERF1* was downregulated (Fig. 2d–f). By contrast, the same expression of *PR-1* and *PDF1.2* as found in wild-type plants was observed in GC26 plants (Supplementary Fig. S4), thus demonstrating that defence gene regulation observed in GC6 and GC27 lines was effectively due to constant high cGMP content. It is worthwhile mentioning that both lines display normal growth of both roots and leaves compared to wild-type plants (Supplementary Fig. S5). Moreover, these datasets demonstrate that transgenic lines GC6 and GC27 lines show reproducible phenotypes and therefor further molecular characterization was carried on only with the line GC6 (hereafter GC).

Together, these results suggested that cGMP modulates the cross-talk between hormone-dependent pathways, promoting JA-dependent responses at the expense of SA-dependent and possibly also ethylene-dependent pathways, downstream of SA and JA production. Indeed, the direct treatment of GC plants with both hormones led to the same altered regulation of *PR-1* and *PDF1.2* in response to salicylic acid or jasmonic acid, respectively, whereas by contrast, high cGMP level seems not to affect ethylene-induced downstream response (Supplementary Fig. S6).

### High cGMP levels alter the cellular redox state in response to avirulent pathogens

**Figure 3:**
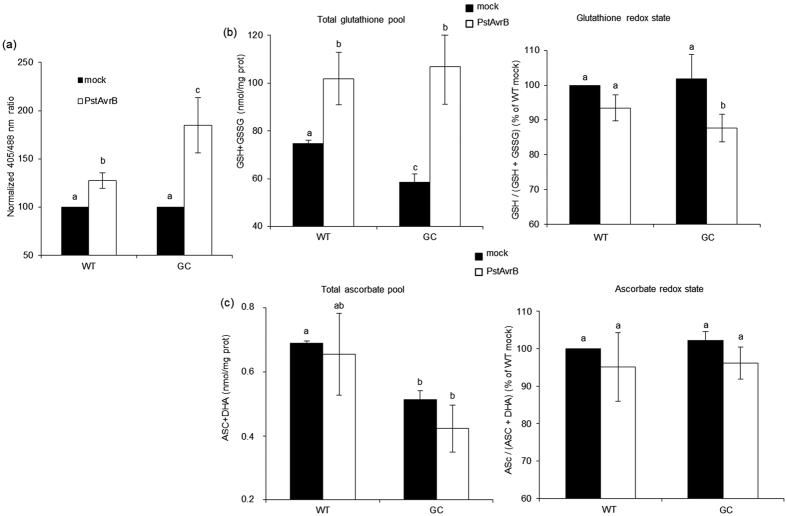
High levels of cGMP alter the glutathione and ascorbate pools as well as the glutathione redox state following infection with *PstAvrB*. (**a**) *A. thaliana* Col-0 and GC plants expressing the genetically encoded GRX-roGFP2 probe were infiltrated with *PstAvrB* (10^7^ cfu/mL). Entire detached inoculated leaves were then observed by fluorescent microscopy 24 hpi and fluorescence emission was evaluated for each condition. Fluorescence values obtained for infected wild-type and GC plants were normalized respect to the values obtained for mock samples, which were fixed to 100 in each biological replicate. (**b,c**) *A. thaliana* Col-0 and GC plants were infiltrated with *PstAvrB* (10^7^ cfu/mL). The total glutathione pool and GSH/(GSH + GSSG) ratio (**b**) as well as total ascorbate pool and ASC/(ASC + DHA) ratio (**c**) were measured 24 hpi. Values obtained for GSH/(GSH+GSSG) and ASC/(ASC+DHA) ratio were normalized respect to the values obtained for mock-infiltrated wild-type samples, which were fixed to 100 in each biological replicate. In each experiment, mock-infiltrated plants were used as controls. Values are means ± SD of three biological replicates. Different letters indicate a statistical difference (p < 0.05) according to Student’s *t*-test (**a**) or ANOVA (**b,c**). GSH, reduced glutathione; GSSG, oxidized glutathione; ASC, ascorbate; DHAR, dihydroascorbate.

NPR1 (non-expresser of PR gene 1) is a master redox sensor^33^. Accordingly, the chemical reduction of NRP1 and thus the induction of NPR1-dependent genes such as *PR-1* correlates with the reductive phase of the defence response^34^. TGA factors have also been postulated as redox sensors^35^ because cysteine modification can modulate their binding to NPR1 and to DNA^36^,^37^. Based on the dysregulation of the NPR1-dependent gene *PR-1* in infected GC plants, we assessed the *in planta* redox state in wild-type and GC line following infection with the avirulent pathogen *PstAvrB*. We generated wild-type and GC plants expressing the genetically-encoded GRX-roGFP2 probe, which specifically reports the ratio between the reduced and oxidized forms of glutathione (GSH/GSSG)^38^. Probe lines were infected with avirulent *PstAvrB* (10^7^ cfu/mL) and mock infiltrations were used as a control. This analysis revealed a clear increase in the fluorescence signal (indicative of glutathione pool oxidation) in both genotypes 24 hpi, but the increase was much more evident in the GC line, indicating a higher oxidative environment in the plants accumulating cGMP (Fig. 3a).

Glutathione and to a lesser extent ascorbate play important roles as small-molecule redox couples during stress-related redox signalling in plants^35^. We therefore measured the total glutathione and ascorbate pools, their redox states, as well as enzyme activities related to the ascorbate–glutathione cycle. Figure 3b shows that the total glutathione pool was similar in the wild-type and GC plants 24 hpi with the avirulent pathogen *PstAvrB* (10^7^ cfu/mL). However, the total glutathione pool declined slightly in the GC line compared to wild-type controls following the mock infiltration, indicating a diminished basal protective state. The glutathione redox state was lower in GC plants than wild-type controls 24 hpi with *PstAvrB*, confirming the results obtained using the GRX-roGFP2 probe (Fig. 3a,b). Accordingly, glutathione reductase (GR) activity detected in the infected GC plants was lower than in wild-type controls following infection with avirulent *PstAvrB* (Supplementary Fig. S7). This change in GR activity was accompanied by a decline in the activity of dehydroascorbate reductase (DHAR), which requires GSH as an electron donor, under the same conditions. Surprisingly, given the loss of DHAR activity in the GC plants, there was no change in the ascorbate redox state (Fig. 3c) and no modulation of ascorbate peroxidase (APX), catalase (CAT) or monodehydroascorbate reductase (MDHAR) (Supplementary Fig. S7). In contrast, high cGMP levels in GC plants caused the total ascorbate pool in the basal state to decline compared to the level observed in wild-type plants. This difference was highlighted by infection with *PstAvrB*, which shrank the total ascorbate pool by ~40% in infected GC plants compared to wild-type controls (Fig. 3c).

### High cGMP levels affect basal and post-infection gene expression profiles

The ratio of reduced/oxidized small-molecule redox couples may play an important signalling role during stress responses, particularly the regulation of gene expression via redox-sensitive mediators^39^. Given the altered glutathione redox state and altered expression of hormone-dependent genes in line GC compared to wild-type plants, we sought deeper insight into the molecular changes correlating with cGMP accumulation by large-scale RNA-Seq analysis.

**Figure 4:**
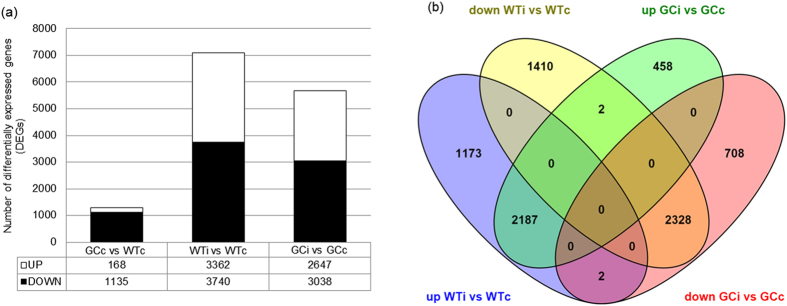
High levels of cGMP modulate both basal and *PstAvrB*-induced gene expression. (**a**) Number of differentially expressed genes (DEGs) in GC plants compared to wild-type plants under basal conditions (GCc, WTc) and following infection with *PstAvrB* (10^7^ cfu/mL) (GCi, WTi). The white bars represent upregulation and the black bars represent downregulation. (**b**) Venn diagram showing the number of unique and common DEGs (induced or repressed) in GC and wild-type plants following infection with *PstAvrB*. The Venn diagram was generated using VENNY (http://bioinfogp.cnb.csic.es/tools/venny/index.html).

Transcriptional changes induced by avirulent *PstAvrB* in GC and wild-type plants were determined by comparing inoculated samples (WTi and GCi) at 12 hpi with uninoculated control samples (WTc and GCc). RNA-Seq analysis was performed with three biological replicates for each condition, with each sample corresponding to a pool of leaves from three different plants. This analysis revealed 7102 differentially expressed genes (DEGs) in infected wild-type plants and 5685 DEGs in infected GC plants with approximately equal numbers of upregulated and downregulated genes in each group (Fig. 4a). It is worth mentioning that a modulation of *PR-1* and *PDF1.2* expression was observed between GC and WT plants following infection, thus confirming the validity of the dataset, according to previous results obtained by real-time PCR (Fig. 2d,e). The comparison of datasets representing GC and wild-type plants highlighted a strong similarity between the genotypes, with 4519 common DEGs compared to 2583 unique DEGs in wild-type plants and 1166 unique DEGs in GC plants (Fig. 4a). Furthermore, the common DEGs were predominantly regulated in a similar manner in both lines – only four genes were regulated in opposite directions in the wild-type and GC plants following infection (Fig. 4b) – and the common DEGs were mainly related to cellular (25%) or metabolic (21%) processes and stress responses (8%) (Supplementary Fig. S8a). A similar distribution of functional categories was also observed for unique DEGs in the infected wild-type (Supplementary Fig. S8b) and GC plants (Supplementary Fig. S8c).

**Figure 5:**
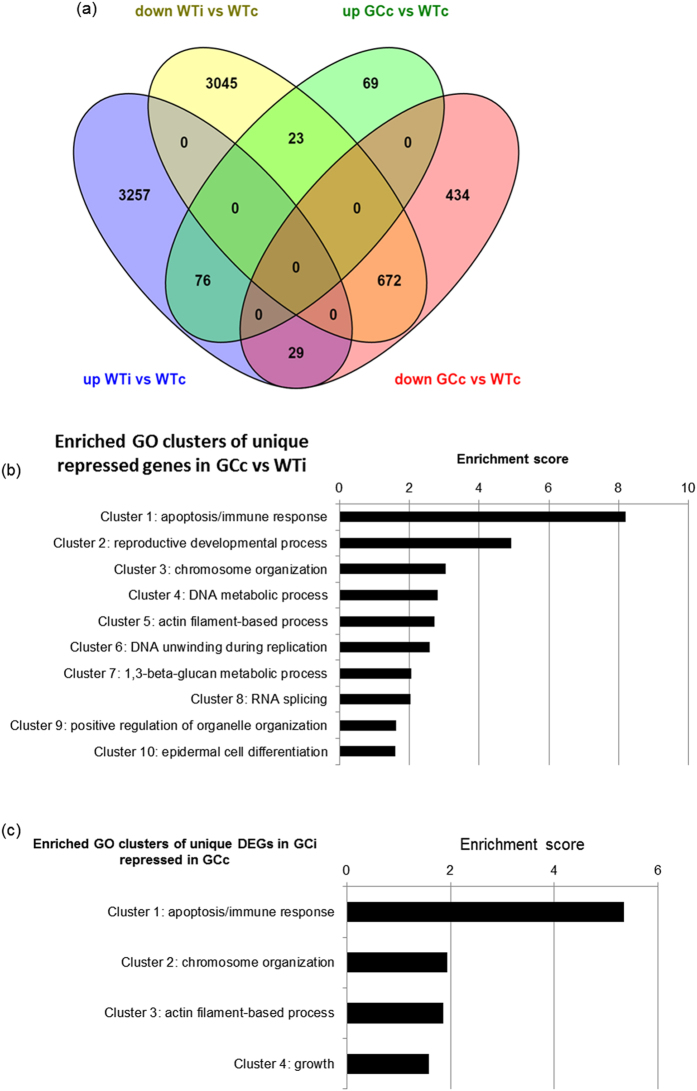
The infection of GC plants with *PstAvrB* abolishes many differences in basal gene expression between GC and wild-type plants. (**a**) Venn diagram showing the number of common and unique DEGs (induced or repressed) under basal conditions in GC plants or in infected wild-type plants. The Venn diagram was generated using VENNY (http://bioinfogp.cnb.csic.es/tools/venny/index.html). (**b**) Clusters of GO categories overrepresented in DEGs that are downregulated in GC plants under basal conditions but not in infected wild-type plants, relative to the whole genome. (**c**) Clusters of GO categories overrepresented in DEGs that are downregulated in GC plants under basal conditions but upregulated uniquely in infected GC plants relative to the whole genome. Cluster enrichment was analyzed using DAVID (https://david.ncifcrf.gov/tools.jsp).

The comparison between uninoculated wild-type and GC samples revealed 1303 DEGs, 87% of which were downregulated by high levels of cGMP (Fig. 5a). Interestingly, among cGMP-repressed genes (GCc vs WTc), 672 genes (52%) were similarly down-regulated in WT plants challenged with *PstAvrB* (WTi vs WTc) and 76 cGMP-upregulated genes (GCc vs WTc) were similarly induced in wild-type following infection (WTi vs WTc) (Fig. 5a), suggesting that GC plants are somehow “primed” for transcriptomic changes related to infection with an avirulent pathogen. Furthermore, GO categories enriched among the 434 genes downregulated by high cGMP level (and not modulated in infected wild-type plants) mainly reflected immune responses and cell death (Fig. 5b). Interestingly, ~54% of these genes were uniquely induced in the infected GC plants, and accordingly the main enriched GO category was again related to immune responses and cell death (Fig. 5c). These results indicate that GC plants in the basal state share part of the transcriptome profile observed in infected wild-type plants, whereas the infection of GC plants with avirulent pathogens abolishes many of the transcriptional differences between the genotypes observed in the basal state, resulting in a gene expression profile similar to wild-type plants.

**Figure 6:**
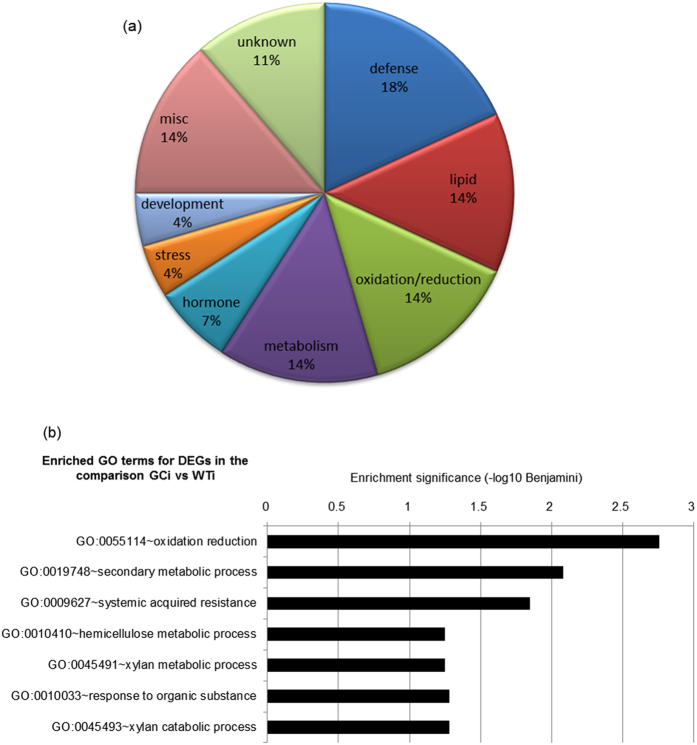
Infected GC plants show the modulated expression of genes related directly or indirectly to SAR. (**a**) Functional category distribution of downregulated genes in GC plants after infection compared to infected wild-type plants. (**b**) GO categories overrepresented in DEGs that are downregulated in infected GC plants compared to infected wild-type plants. GO category enrichment was analyzed using DAVID (https://david.ncifcrf.gov/tools.jsp).

To highlight the retained transcriptome differences between the genotypes by overcoming the effect of infection, we analysed DEGs in infected GC plants compared to infected wild-type plants, which resulted in 146 DEGs (88 downregulated and 58 upregulated). Despite the global similarity between transcriptomes, the distribution of functional categories among the genes less expressed in GC plants compared to wild-type indicated the modulation of genes related to defence (18%), lipid metabolism (14%), oxidation/reduction (14%) and general metabolism (14%) (Fig. 6a). We therefore enlarged the analysis by including DEGs displaying a log2FC ≥ 0.5 or log2FC ≤ −0.5 (p ≤ 0.05). Interestingly, the enriched GO categories mirrored the distribution observed among DEGs with log2FC ≤ −1 with a significant enrichment of genes involved in pathways related to oxidation/reduction, secondary metabolic processes and defence, particularly SAR (Fig. 6b). The schematic representation of detailed overrepresented categories in each pathway revealed the significant downregulation of fatty acid metabolism and lipid localization (Supplementary Fig. S10 and Supplementary Table S2), and responses to hormone stimuli (Supplementary Fig. S10 and Supplementary Table S3). Interestingly, most SAR-related genes were involved in SAR signal production, e.g. *FMO1*, *aza1* and *MES9* (Table 1).

### High cGMP levels modulate the protein profile following infection with avirulent *PstAvrB*

We also analysed the leaf proteome to complete the catalogue of molecular changes occurring in GC plants with high levels of cGMP. Samples were prepared 24 hpi to complement the transcriptomic analysis described above. Moreover, as for RNA-Seq experiment, proteomic analysis was performed with three biological replicates for each condition, with each sample corresponding to a pool of leaves from three different plants.

**Figure 7:**
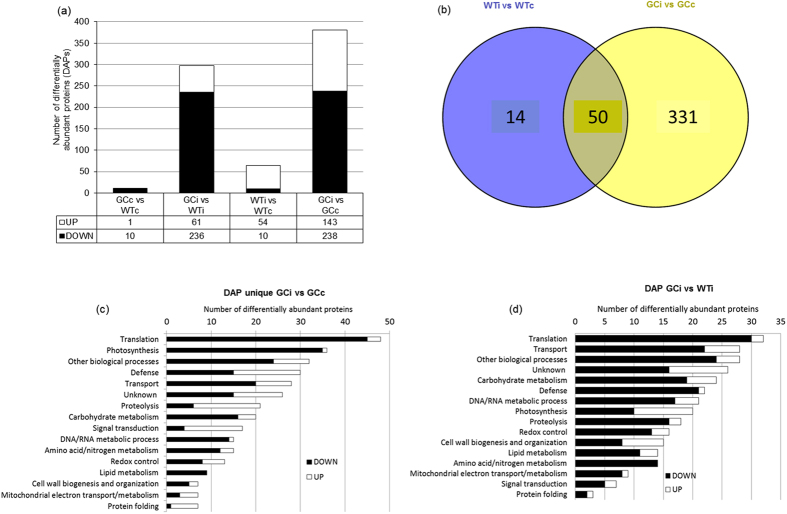
Proteomic analysis reveals the strong modulation of protein levels in *PstAvrB*-infected GC plants compared to wild-type controls. (**a**) The number of differentially abundant proteins in GC plants compared to wild-type plants under basal conditions and following infection with *PstAvrB* (10^7^ cfu/mL). The white bars represent upregulation and the black bars represent downregulation. (**b**) Venn diagram showing the number of unique and common differentially abundant proteins in GC and wild-type plants following infection with *PstAvrB* (10^7^ cfu/mL). (**c**) Functional category distribution of unique differentially abundant proteins following the infection of GC plants with *PstAvrB* (10^7^ cfu/mL). (**d**) Functional category distribution of differentially abundant proteins in infected GC plants compared to infected wild-type plants.

Figure 7 shows the major proteomic differences between GC and wild-type plants in the basal state and following infection. Constitutively high levels of cGMP had a minor impact on the basal proteome, resulting in only 11 differentially abundant proteins (DAPs) when the GCc and WTc groups were compared (Fig. 7a and Supplementary Table S4). In contrast, we observed the substantial modulation of the proteome in GC plants compared to wild-type controls following infection with avirulent *PstAvrB*, with 64 and 381 DAPs observed in the WTi and GCi groups, respectively (Fig. 7a), including 50 proteins common to both genotypes (Fig. 7b). Most of the DAPs in the GCi group were downregulated (Fig. 7a). Several unique DAPs related to defence functions accumulated only in the infected wild-type plants (Supplementary Table S5), whereas the 331 unique DAPs found in infected GC plants included functional categories related to translation (15%), photosynthesis (11%), defence (8%) and transport (8%) (Fig. 7c). Taking into account these substantial differences in proteome modulation following infection, we compared the DAPs in the GCi and WTi groups (Fig. 7d) to highlight the effect of the genotype over infection, revealing that the main functional categories were related to translation (11%) and transport (9%). However, 7% of the DAPs were related to defence responses and most (95%) of the proteins within this functional category were downregulated (Table 2). Whereas several of these DAPs are positively related to defence responses, such as the cinnamyl-alcohol dehydrogenase 7 an elicitor-responsive enzyme involved in lignin biosynthesis, the two phospholipases D PLDα1 and PLDδ, which play an important role in lipid-mediated defences or the GSNO reductase responsible for S-nitrosoglutathione level regulation, to name a few, it is worth noting that by contrast some downregulated proteins are involved in the negative regulation of plant defences, such as the cytosolic glyceraldehyde-3-phosphate dehydrogenases (GAPC1) and the Arabidopsis Nudix hydrolase homolog 7 (AtNUDT7), the level of which decreased by log2FC −1,23 and −4,94, respectively.

The analysis of GO category enrichment allowed us to focus on the main pathways affected in the infected GC plants, confirming that the high cGMP levels strongly repress pathways involved in translation, protein transport and protein localization (Supplementary Fig. S10). Other categories overrepresented among the downregulated proteins in infected GC plants represented abiotic stress responses and metabolic processes (Supplementary Fig. S10). These data indicate that cells with high levels of cGMP respond to infection mainly by adapting primary metabolism and protein synthesis/transport. It is worth mentioning however that proteomics studies are highly biased towards high abundant proteins (ribosomes, metabolic enzymes), thus leading to an underrepresentation of regulatory components, such as kinases or transcription factors for example, which might also explain the lack of significant changes in these categories pointing towards pathogen infection.

### SAR induced by avirulent *PstAvrB* is compromised by the accumulation of cGMP

**Figure 8:**
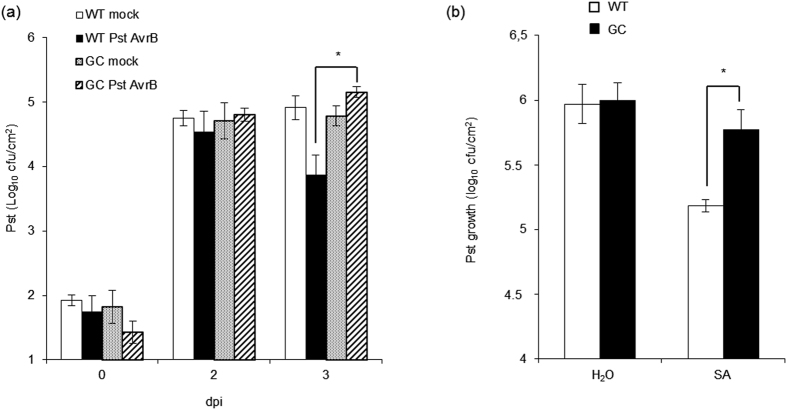
SAR induced by *PstAvrB* and salicylate is compromised in plants accumulating cGMP. Two fully-expanded *A. thaliana* Col-0 or GC leaves were infiltrated (primary inoculation) with *PstAvrB* (10^7^ cfu/mL) (**a**) or salicylic acid (1mM) (**b**). Mock-infiltrated (**a**) or water-infiltrated (**b**) plants were used as controls of *PstAvrB*-induced or SA-induced SAR, respectively. In each experiment, the upper uninfected leaves were then infiltrated with virulent *Pst* (2.5 × 10^5^ cfu/mL). Data are expressed as means of three replicates ± SD. Experiments were carried out three times with similar results. Asterisks indicate a statistical difference (p < 0.05) according to ANOVA test.

Our transcriptomic data indicated that SAR-related genes are downregulated in GC plants, so we investigated whether these plants could achieve normal SAR following infiltration with avirulent *PstAvrB*. Three lower rosette leaves of GC and wild-type plants were infiltrated either with 10 mM MgCl_2_ (mock infiltration) or with a suspension of *PstAvrB* (10^7^ cfu/mL) to induce SAR. Two days later, three upper (untreated) leaves were challenged with virulent *Pst* (2.5 × 10^5^ cfu/mL). SAR was assessed by scoring bacterial growth in the upper leaves 3 days after the second infection. Wild-type plants pre-treated with *PstAvrB* achieved significantly enhanced resistance against *Pst* infection compared to the mock-infiltrated controls, displaying lower bacterial growth (Fig. 8a). In contrast, the GC line completely failed to achieve SAR in response to a primary *PstAvrB* infection (Fig. 8a). We then investigated whether SAR was blocked upstream or downstream of the inducer SA by treating wild-type and GC plants with 0.5 mM SA 2 days before infecting the upper leaves with virulent *Pst* (2.5 × 10^5^ cfu/mL). Three days later, SAR was again assessed by scoring bacterial growth. Once again, the SA-treated wild-type plants displayed greater resistance than control plants, but the GC plants failed to achieve SAR in response to SA treatment (Fig. 8b). This confirms that SAR in the GC transgenic plants is blocked downstream of SA synthesis.

**Figure 9:**
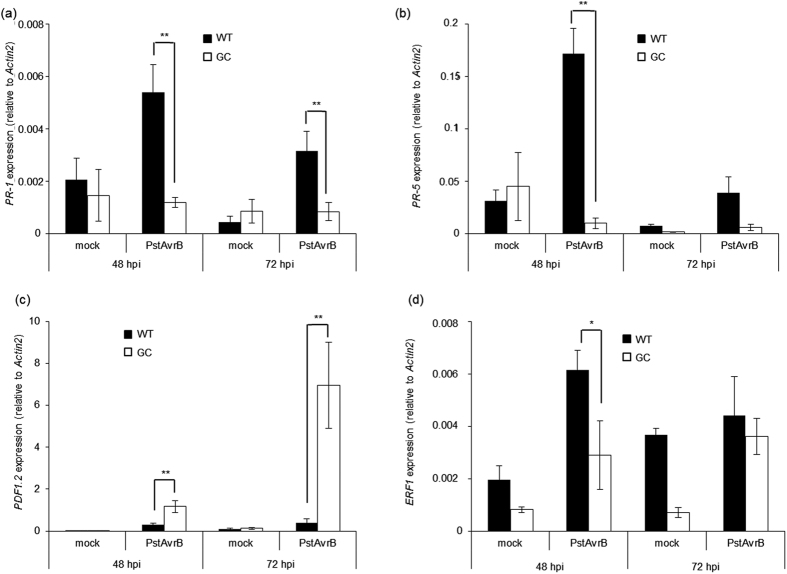
High levels of cGMP alter hormone-dependent gene expression in response to *PstAvrB* at a systemic level. *A. thaliana* Col-0 and GC plants were infiltrated with *PstAvrB* (10^7^ cfu/mL) and distal uninfected leaves were collected 48 and 72 hpi for the analysis of *PR-1* (**a**), *PR-5* (**b**), *PDF1.2* (**c**) and *ERF1* (**d**) expression by real-time RT-PCR using gene-specific primers. The expression level of each gene was normalized to that of *Actin2*. In each experiment, mock-infiltrated plants were used as controls. Values are means ± SEM of at least three biological replicates. Asterisks indicate a statistical difference (*p < 0.1; **p-value < 0.05) according to ANOVA test.

At the molecular level, defence gene expression was analysed in the upper leaves of wild-type plants following the infection of lower leaves with avirulent *PstAvrB*, confirming the significant induction of the expression of the SA-dependent genes *PR-1* and *PR-5* at 48 and 72 hpi, but only weak induction of the ethylene-dependent gene *ERF1* and no change in the jasmonate-dependent gene *PDF1.2*, in each case compared to the mock infiltration controls (Fig. 9). In contrast, *PR-1* and *PR-5* were significantly repressed in GC plants compared to wild-type plants following infection (Fig. 9a,b), whereas *PDF1.2* was strongly upregulated (Fig. 9c), suggesting the possible interruption of systemic SA–JA cross-talk in plants accumulating high levels of cGMP. *ERF1* expression was also significantly reduced in the GC line compared to wild-type plants (Fig. 9d), indicating a possible inhibition also of ethylene-dependent pathway.

## Discussion

The role of cGMP in plant defence responses is difficult to study in detail because the enzymes responsible for its synthesis and degradation are currently unknown. We therefore created transgenic *A. thaliana* plants expressing the rat soluble guanylate cyclase (GC) to produce a line that accumulates high levels of cGMP, which can thus be used to study cGMP-dependent cellular responses. Similarly, the cardiomyocyte-specific expression of the constitutive GC domain of the NPRA receptor in mice led to an increase in cGMP levels^40^. Surprisingly, although the rat soluble GC is inducible by NO in animal cells^41^, our transgenic *A. thaliana* GC lines accumulated high cGMP levels without induction, indicating that the enzyme is constitutively active *in planta*. The haem moiety coordinated by His-105 in the beta subunit maintains the regulatory domain in a restricted basal conformation, so we propose that the recombinant enzyme and haem prosthetic group adopt a different conformation in plant cells that favours constitutive activity^41^. Furthermore, because the intracellular accumulation of cGMP reflects the balance between GC-dependent synthesis and degradation by PDEs, we also propose that the amount of cGMP produced by the GC transgenic lines is so high (50–250-fold higher than normal) that the relatively low endogenous PDE activity^29^ is saturated, thus shifting the GC/PDE ratio in favor of cGMP accumulation. Likewise, Sf9 cells expressing a mutant haem-deficient enzyme accumulate high levels of cGMP, which cannot be degraded efficiently by endogenous PDEs^42^. Accordingly, cGMP accumulation in our GC lines remains unchanged following infection with pathogens (virulent and avirulent), indicating that the GC lines have lost the ability to modulate cGMP synthesis in response to pathogens and that endogenous PDE activity is insufficient to remove cGMP from the transgenic plants. Such hypothesis is further supported by the transgenic GC line 26, which displays the lowest cGMP accumulation compared to the other two transgenic lines and the cGMP level of which seems to be still controlled by endogenous metabolism, at least following pathogen infection.

Despite the significant basal downregulation of defence-related genes in the GC lines, particularly genes involved in programmed cell death, no differences in phenotype were observed in terms of local responses, *i.e.* resistance to virulent or avirulent pathogens and hypersensitive cell death. This may reflect the significant upregulation of some of these genes following infection, restoring the expression levels observed in infected wild-type plants. Accordingly, several defence-related DEGs that were downregulated in the GC lines under basal conditions were upregulated following infection (DEGs uniquely upregulated in the GC lines) and ultimately returned to the expression levels observed in wild-type plants. Moreover, proteomic analysis revealed the stronger downregulation of several negative defence regulators in infected GC plants compared to infected wild-type plants, which may compensate for the weaker defence response in GC plants and yield a normal resistance phenotype. For example, the amount of GAPC1 protein (At3g04120) declined sharply in GC plants following infection (compared to wild-type plants), and *gapc1* mutants likewise display a stronger resistance phenotype and accelerated cell death compared to wild-type plants^43^. Similarly, the NUDIX protein (AtNUDT7), a negative regulator of basal immunity^44^, accumulated to lower levels in infected GC plants compared to wild-type plants. Interestingly, *nudt7* mutants have a lower GSH/GSSG ratio and therefore an altered redox state following infection, which may explain the enhanced resistance^44^. A larger oxidized glutathione pool was also observed in the GC lines following infection with avirulent *PstAvrB*, and this could contribute to the normal resistance phenotype by compensating for the suppression of defence-related gene expression. The downregulation of AtNUDT7 may affect the phenotype of the GC lines by sensing and modulating the levels of nucleotide analogues, particularly cGMP^44^.

The higher oxidative redox state observed in the GC plants compared to wild-type controls following infection correlated with a decline in the activities of relevant enzymes after infection, namely glutathione reductase (GR) and dihydroascorbate reductase (DHAR), suggesting that cGMP accumulation alters the glutathione cycle. Since no significant changes in genes or proteins regulating the production or removal of ROS or more generally controlling cellular redox state was identified in transcriptomics and proteomics experiments, we can assume that the regulation of these enzymes, and others playing a role in the regulation of redox homeostasis, occurs at post-translational level. This could be achieved by the direct binding of cGMP to target enzymes, as recently reported in Arabidopsis for enzymes involved in Calvin cycle and photorespiration, such as the glycolate oxidase 1 (GOX1)^45^, or indirectly by the regulation of signalling components such as cGMP-dependent protein kinases (PKG), as in animals^46^. Accordingly, phosphorylation was shown to control the activity of the potato NAPDH oxidase RBOHB^47^. The more oxidized state, particularly the reduction of GR activity and GSH, could be partially responsible for the observed downregulation of SAR-related genes, such as *PR-1* and *PR-5*. Indeed, a reducing environment (particularly reduced GSH) is required for NPR1 activation (monomer formation) and translocation to the nucleus^34^,^48^. Thus the more oxidative environment observed in GC plants may help to maintain NPR1 as an inactive oligomer thus blocking downstream NPR1-dependent gene expression. In agreement with this hypothesis, we observed that high cGMP levels block SAR downstream of SA, as shown by the inability of the transgenic plants to achieve SAR even when treated directly with SA. In contrast, any perturbation in the redox balance may “prime” cells to respond more aggressively to pathogen infection, as shown in the *nudt7* mutant^44^. In this scenario, the oxidative status of the GC line although slightly different from WT plants but statistically significant in both approaches, could be sufficient to explain the impairment of SAR downstream of SA while accounting for the normal resistance phenotype, as an alternative mechanism for compensating for the downregulation of immunity-related genes and proteins.

In addition to *PR* gene expression, other genes related to SAR were downregulated in the GC line following infection with avirulent *PstAvrB*. Two functional categories of genes were significantly overrepresented among the DEGs when GC and wild-type lines were compared, namely those relating to hormone regulation and lipid metabolism, both involving key regulators of SAR^49^. We found that SA-dependent genes were predominantly downregulated whereas most JA-dependent genes were upregulated, suggesting that high cGMP levels affect the SA/JA regulatory balance. Such regulation likely takes place downstream of hormone biosynthesis according to the fact that the expression of *PR1* and *PDF1.2*, marker genes of SA and JA respectively, is also altered in GC plants directly treated with the hormones. Some DEGs were regulated by ethylene, abscisic acid (ABA), auxins, cytokinins or brassinosteroids, which also play a role in plant–pathogen interactions^50^. Interestingly, an increase in cGMP levels has been observed in response to ABA, auxins, JA and brassinosteroids, indicating a role for cGMP downstream of these hormonal signals^51^,^52^. Together these data suggest that cGMP could exert feedback control on the responses mediated by these phytohormones. We also noticed that all ethylene-response factors were downregulated in the GC lines, indicating the negative regulation of these transcription factors in the presence of high levels of cGMP. Ethylene receptor 1 (ETR1) contains a GAF domain, which binds cGMP and mediates light regulation, but the precise function of ETR1 remains unknown^53^,^54^. It would be interesting to investigate whether cGMP suppresses ETR1 by binding to its GAF domain, leading to the repression of ethylene-responsive factors required to establish SAR.

In agreement with the hypothesis described above, ethylene perception may be involved in the generation and/or release of the mobile signal that induces SAR in non-infected tobacco tissues^55^. Accordingly, some of the downregulated genes identified during the transcriptomic analysis of the GC plants mediate SAR signal production, particularly lipid transfer proteins (LTPs) that help to establish SAR in a SA-independent manner^56^. Interestingly, mammalian LTPs can act as lipid sensors or mediate signal transduction pathways dependent on phospholipase C^57^. The non-specific LTP DIR1 (Defective in induced resistance 1) is defective in the production or transmission of an essential mobile signal that probably interacts with a lipid-derived molecule to promote long-distance signalling^58^.

Phospholipase D (PLD) and PLD-derived phosphatidic acids are another class of lipid-related molecules that have a fundamental impact on almost all hormone signalling pathways^59^. For example, PLDα1 must be activated downstream of SA^60^,^61^ and PLDα1-PA regulates ABA signalling^62^. Interestingly, PLDα1 was downregulated at the protein level in infected GC plants compared to infected wild-type plants, creating a possible link between the modulation of PLD by cGMP and a phytohormone imbalance that blocks SAR. PLDδ is another defence-related PLD and this was also downregulated in infected GC plants. PLDδ interacts with two cytosolic glyceraldehyde-3-phosphate dehydrogenases (GAPC1 and GAPC2) to regulate lipid metabolism^63^ and it is worth noting that GAPC1 protein accumulation was also downregulated in infected GC plants compared to wild-type counterparts. This suggests that cGMP accumulation in the GC line affects the PLDδ-PA-GAPC1 interaction complex, leading to the downregulation of lipid metabolism indicated by our transcriptomic data. Once again, the modulation of this pathway could help to prevent the GC plants from achieving SAR. Finally, in the SA-independent branch of SAR induction, NO and ROS act directly upstream of the dicarboxylic acid azelaic acid^6^,^64^. We found that *AZA1* was repressed in the infected GC line compared to wild-type plants, and we confirmed that cGMP acts downstream of NO, thus placing cGMP in this pathway. Moreover, the normal NO accumulation observed in transgenic GC plants both at basal level and following infection with avirulent bacterial pathogen (Supplementary Figure 11) ensures that SAR impairment in cGMP-accumulating plants is not due to a higher NO production, known to alter SAR establishment^6^. However, high cGMP levels also affect SA-dependent responses given that the establishment of SAR is also impaired when SA is applied directly. We therefore propose a direct role for cGMP in the control of azelaic acid-mediated SAR, and an indirect role involving the regulation of phytohormone cross-talk. Moreover, the alteration of the redox state by high cGMP content could also influence SAR by impairing the signals mediated by NO, ROS and SA. Nevertheless, we do not rule out the possibility that cGMP could also play a role at systemic level according to the accumulation of the second messenger in distal leaves following infection with *PstAvrB* (Supplementary Fig. S12).

Overall, the characterization of our GC line favours a negative role for cGMP downstream of NO in the regulation of plant defences, in particular SAR, which fits neither with the function of NO in plant defence signalling nor with the putative role proposed for cGMP so far (although this is a general role rather than a specific role in SAR). In particular, it was reported very recently that leaf injury induced the systemic accumulation of cGMP in pumpkin extrafascicular phloem that could function in systemic NO and redox signalling within the EFP as positive defence mechanism against herbivorous^65^. The key feature of the GC line was the constitutive high level of cGMP compared to wild-type plants. However, second messengers such as cGMP usually work by transient accumulation^66^ and this transient aspect is completely abolished in the GC plants. We therefore propose that the high constitutive cGMP levels in the GC lines could desensitize the plants to cGMP stimuli, i.e. the biphasic variations required for normal cGMP signal perception and responses. In agreement with this hypothesis, the comparison of DEGs in plants constitutively accumulating cGMP (this study) or in roots treated with a bolus of the analogue 8-Br-cGMP for 2 or 5 h^67^, reveal only a slight overlap between the datasets (Supplementary Fig. S13). Interestingly, most of the common DEGs in these datasets are diametrically regulated, indicating that the constitutive high cGMP levels in our GC lines suppress genes that are normally induced by transient cGMP spikes (8-Br-cGMP or transient increases observed during the HR). Accordingly, the rapid desensitization of GC in animal cells, in concert with variations in the rate of cGMP degradation, provides a fundamental mechanism for shaping cellular cGMP responses and this is likely to facilitate the decoding of NO signals under both physiological and pathophysiological conditions^68^,^69^. Furthermore, silencing the rod PDEγ in W70A mutant mice leads to cGMP accumulation, which is responsible for a loss-of-function phenotype involving a desensitized response to light^69^.

We have demonstrated the first genetic approach to study the role of cGMP in plant defence responses by expressing mammalian soluble GC in *A. thaliana* plants. The characterization of local responses of transgenic GC plants accumulating more than 50-fold more cGMP revealed normal cell death and resistance. In contrast, the modulation of gene expression and protein abundance at the local level had a negative impact on the establishment of SAR in GC plants challenged with an avirulent pathogen, with a strong effect on hormone-related responses and lipid metabolism and transport. These results represent the first evidence of the participation of cGMP in plant systemic responses induced by bacterial infection, acting in particular at local infection level by regulating SAR signal production and transport. Moreover, the use of such cGMP-accumulating transgenic plants creating a possible model of cGMP desensitization indicated that cGMP effect is likely mediated not strictly by its absolute concentration but rather its biphasic variations. The analysis of SAR in plants depleted for cGMP, e.g. plants overexpressing PDE, will help to confirm further the desensitization of GC lines to cGMP signals to confirm that cGMP-mediated responses require such precise transient biphasic variation to control the signals that induce SAR.

## Methods

### Plant material and growth conditions

The *A. thaliana* plants used in this study were ecotype Columbia (Col-0) and a line expressing the GRX-roGFP2 probe was already available^38^. Plants were grown in a growth chamber with a short day cycle of white light (~100 μE), 24 °C/22 °C day/night temperature and 70% relative humidity.

For the selection of transgenic plants, seeds were surface sterilized and plated in Petri dishes on half-strength Murashige & Skoog (MS) medium containing 0.8% agar (Duchefa Biochemie, Haarlem, Netherlands) and appropriate antibiotics. After cold-treatment for 2–3 days, the plates were transferred to the growth chamber, and seedlings were transferred to soil when they were 10–12 days old.

### Generation of *A. thaliana* plants expressing mammalian soluble guanylate cyclase

The alpha and beta subunits of mammalian soluble guanylate cyclase (GC) (GenBank M57405 and M22562) were previously cloned as cDNAs from rat lungs an transferred to a mammalian expression vector^71^,^72^. These vectors were kindly provided by Dr M Nakane, Chicago Futabakai Japanese School, Chicago, Illinois. Both genes were transferred to a plant expression vector containing the CaMV 35S promoter using Gateway technology (Invitrogen, Thermo Fisher Scientific, Waltham, Massachusetts, USA). This involved the initial transfer of each gene into the pENTR vector (Invitrogen), then the alpha subunit cDNA was transferred to vector pH7WG2, conferring hygromycin resistance, and the beta subunit cDNA was transferred to vector pB7WG2, conferring phosphinothricin resistance, to allow the selection of double transformants based on dual resistance. *A. thaliana* Col-0 plants were co-transformed using the floral-dip method^73^ with a 1:1 ratio of two strains of *Agrobacterium tumefaciens* GV3101 containing each vector. Seeds were surface sterilized and plated on half-strength MS medium including 0.8% agar and the antibiotics needed for dual selection.

### Generation of *A. thaliana* GC plants expressing the GRX-roGFP2 probe

The GC transgenic line described above was super-transformed using the floral-dip method^73^ with *A. tumefaciens* strain GV3101 carrying the GRX-roGFP2 construct under the control of the UBQ10 promoter^38^,^74^. Seeds were surface sterilized and plated on half-strength MS medium including 0.8% agar and the seedlings were screened under a stereomicroscope equipped with a fluorescent lamp and green fluorescent protein (GFP) filter. Seedlings expressing GFP were isolated and transferred to soil. Seeds from two independent transgenic lines were combined and used for the pathogen challenge experiments. None of the transgenic lines showed an abnormal phenotype under our standard growth conditions.

### Plant infection and treatment

*Pseudomonas syringae* pv. *tomato* DC3000 (*Pst*) and *Pst* DC3000 carrying the avirulence gene *AvrB* (*PstAvrB*) were grown overnight at 28 °C in King’s B medium containing the appropriate antibiotics (50 mg/L rifampicin, 50 mg/L kanamycin). Bacteria were pelleted, washed three times with 10 mM MgCl_2_, resuspended, and diluted in 10 mM MgCl_2_ to the desired concentration (generally 10^7^ cfu/mL, but 5 × 10^5^ cfu/mL for bacterial growth assays). The bacterial solutions were infiltrated from the abaxial side into leaves using a 1-mL syringe without a needle. Control (mock) inoculations were carried out with 10 mM MgCl_2_. For transgene induction, *hmpX* plants were sprayed with a solution of 3 mM dexamethasone (DEX) in 0.01% Tween-20 24 h before pathogen infiltration.

### Analysis of cell death

The HR electrolyte leakage assay was carried out as previously described^75^. Leaf discs (8 mm diameter) were vacuum-infiltrated with bacterial suspensions (1 × 10^7^ cfu/mL), rinsed in water for 30 min and then transferred to Petri dishes containing 6 mL Milli-Q water, and conductance was measured over for 24 h using a B-173 compact conductivity meter (Horiba Ltd, Kyoto, Japan).

### Analysis of bacterial growth and systemic acquired resistance

Bacterial growth kinetics in *A. thaliana* leaves was investigated by infiltrating the leaves with 2.5 × 10^5^ cfu/mL of bacterial suspension and assaying the growth as previously reported^76^.

### Extraction of cGMP from leaves

Approximately 0.1–0.15 g of *A. thaliana* leaf tissue was collected from wild-type and transgenic GC lines in a 1.5-mL Eppendorf tube containing glass beads. The tubes were frozen in liquid nitrogen and ice-cold 6% trichloroacetic acid (TCA) was added before mechanical grinding. The homogenate was centrifuged three times (10,000 × *g*, 5 min, 4 °C) and the aqueous supernatant was washed four times with five volumes of water-saturated diethyl ether on ice. Finally, the samples were frozen in liquid nitrogen and lyophilized overnight.

### AlphaScreen cGMP assay

A biotinylated cGMP supplement containing an anti-cGMP antibody, protein A-coated acceptor beads and streptavidin-coated donor beads was purchased from Perkin Elmer Corp. (Waltham, Massachusetts, USA). Biotinylated cGMP was prepared at a concentration of 5 nM in assay buffer (25 mM HEPES, pH 7.4, 100 mM NaCl, 0.1% Tween-20) containing 1 mg/mL bovine serum albumin (BSA). The acceptor bead mix was prepared in assay buffer with the anti-cGMP antibody (diluted 1:2000) and protein A-coated acceptor beads (50 μg/mL). Streptavidin-coated donor beads were prepared in assay buffer at a concentration of 100 μg/mL. The cGMP standard was prepared as a serial dilution from 0.5 nM to 5 μM in assay buffer without BSA. Lyophilized samples were resuspended in assay buffer without BSA. The reaction was carried out in white opaque 384-well plates (Optiplate TM-384, Perkin Elmer) with 10 μL of the acceptor bead mix in each well together with 5 μL of cGMP standard dilutions or lyophilized samples. The plates were incubated for 20 min at room temperature in the dark before adding 5 μL of biotinylated cGMP and incubating for a further 5 min at room temperature. Finally, 5 μL of diluted streptavidin-coated donor beads was added and the reaction was incubated for 2 h at room temperature in the dark. Fluorescence was then measured using the Enspire multi-label plate reader (Perkin Elmer).

### Redox imaging

Whole leaves from 5–6-week-old plants expressing the GRX-roGFP2 redox sensor**38, inoculated with 10^7^ cfu/mL avirulent *PstAvrB* or 10 mM MgCl_2_ as a control, were cut at the level of the petiole, gently placed on a slide and covered with a coverslip, and viewed using a Nikon Ti-E inverted fluorescence microscope (Nikon Corp., Tokyo, Japan) with a CFI Plan Fluor 4 × N.A. 0.13 dry objective. The slides were illuminated using a Prior Lumen 200 PRO fluorescent lamp (Prior Scientific, Cambridge, UK) with 4 × 4 CCD binning, switching between 470/40 nm (100 ms exposure) and 405/40 nm (500 ms exposure). Images were collected using a Hamamatsu Dual CCD Camera ORCA-D2 (Hamamatsu Photonics, Hamamatsu City, Japan). The GRX-roGFP2 emissions were collected using a 505–530 nm bandpass filter (Chroma Technology Corp., Bellows Falls, Vermont, USA) at both excitation wavelengths. Images were acquired every 5 s for 1 min. NIS-Elements software (Nikon) was used to control the microscope, illuminator, camera and post-acquisition analysis. Fluorescence intensity was determined over regions of interest corresponding to the entire visual field with no background subtraction. For GRX-roGFP2 analysis, the two emissions covering the region of interest were used to calculate the 405/488 ratios of individual images during the 1-min acquisition interval, and the averaged ratios were normalized to those of the mock-infiltrated plants. The experiments were repeated with three different plants and at least with two different leaves per condition per plant.

### Ascorbate and glutathione assays

Approximately 0.3 g of *A. thaliana* leaf material was collected from wild-type and transgenic GC lines and homogenized with six volumes of 5% meta-phosphoric acid at 4 °C. The homogenate was centrifuged at 20,000 × *g* for 15 min at 4 °C, and the supernatant was collected for the analysis of ascorbate (ASC) and glutathione (GSH) levels and redox states as previously described^77^. ASC and GSH levels were calculated as percentages compared to the mock-infiltrated plants.

### Enzyme assays

Leaves from wild-type and transgenic GC lines were ground in liquid nitrogen and homogenized at 4 °C in four volumes of 50 mM Tris-Cl (pH 7.8), 0.05% (w/v) cysteine, 0.1% (w/v) BSA and 1 mM ASC. The homogenate was centrifuged at 20,000 × *g* for 15 min at 4 °C and the supernatant was analyzed by spectrophotometry. Proteins were quantified using the Bradford method^78^ with BSA as a standard.

The activities of ASC peroxidase (APX) (L-ASC:H_2_O_2_ oxidoreductase, EC 1.11.1.11), monodehydroascorbate reductase (MDHAR) (NADH:ASC free radical oxidoreductase, EC 1.6.5.4), dehydroascorbate reductase (DHAR) (GSH:dehydroascorbate oxidoreductase, EC 1.8.5.1) and GSH reductase (GR) (NADPH:GSH disulfide oxidoreductase, EC 1.6.4.2) were determined as previously described^79^. Catalase (CAT) (H_2_O_2_:H_2_O_2_ oxidoreductase, EC 1.11.1.6) activity was determined as previously described^80^ with minor modifications, by following H_2_O_2_ dismutation at 240 nm in a reaction mixture comprising 0.1 M phosphate buffer (pH 7.0), 50–100 μg protein and 18 mM H_2_O_2_ (extinction coefficient 23.5 mM^−1^ cm^−1^). Enzyme activities were calculated as percentages compared to the mock-infiltrated plants.

### RNA isolation

RNA was isolated using Trizol reagent (Invitrogen) according to the manufacturer’s instructions. RNA quality and quantity were determined using a Nanodrop 2000 spectrophotometer (Thermo Fisher Scientific). Total RNA was purified using the TURBO DNA-free™ Kit (Thermo Fisher Scientific) and first-strand cDNA was synthesized using SuperScript™ II reverse transcriptase (Thermo Fisher Scientific). The cDNA was used for real-time RT-PCR analysis or samples were processed for library preparation.

### Real-time RT-PCR expression analysis

Real-time RT-PCR was carried out using Platinum^®^ SYBR^®^ Green qPCR SuperMix-UDG (Thermo Fisher Scientific) and the *Actin2* housekeeping gene (GenBank AF428330.1). For each gene of interest, the expression has been calculated as the relative transcript abundance respect to the expression of *Actin2* used as reference gene. Three technical replicates for each biological replicate were used for each sample and template-free negative control. Gene-specific primers were designed using Primer3 software and their sequences are shown in Supplementary Table S1.

### Proteomic analysis

Wild-type and transgenic GC lines infected with avirulent *PstAvrB* or mock infiltrated controls were collected at 24 hpi. Three biological replicates for each condition were prepared, where each replicate corresponded to the pool of three leaves coming from three different plants. A detailed description of the procedure is provided in Supplementary Information.

### RNA-Seq analysis

Wild-type and transgenic GC lines infected with avirulent *PstAvrB* or mock infiltrated controls were collected at 12 hpi. Three biological replicates for each condition were prepared, where each replicate corresponded to the pool of three leaves coming from three different plants. Total RNA was extracted as described above and detailed description of RNA-Seq procedure is provided in Supplementary Information.

### RNAseq data deposition

Transcriptome sequence data from this article can be found in the National Center for Biotechnology Information (NCBI) Gene Expression Omnibus under the series entry GSE86471 (http://www.ncbi.nlm.nih.gov/geo/query/acc.cgi?acc=GSE86471).

### Gene ontology

Gene ontology (GO) analysis was carried out for term enrichment using the AgriGO Single Enrichment Analysis tool^81^ with TAIR10 GO annotations and full GO, as well as The Database for Annotation, Visualization and Integrated Discovery (DAVID) v6.7 with TAIR_ID gene lists^82^,^83^. The hypergeometric test was used with Benjamini-Hochberg FDR correction and p < 0.05. Genes expressed at low and higher levels were analyzed separately.

### Statistical Analysis

All data were presented as means ± standard deviation (SD). Statistical analysis was carried out using GraphPad Prism v4.02. One-way analysis of variance (ANOVA) and non-parametric tests were used with p-values determined using Tukey’s comparison test or Student’s *t*-test. Differences were considered statistically significant at p < 0.05.

## Additional Information

**How to cite this article**: Hussain, J. *et al*. Constitutive cyclic GMP accumulation in *Arabidopsis thaliana* compromises systemic acquired resistance induced by an avirulent pathogen by modulating local signals. *Sci. Rep.* **6**, 36423; doi: 10.1038/srep36423 (2016).

**Publisher’s note:** Springer Nature remains neutral with regard to jurisdictional claims in published maps and institutional affiliations.

## Supplementary Material

Supplementary Information

## Figures and Tables

**Table 1:** Genes directly involved in the regulation/establishment of SAR that are downregulated in infected GC plants compared to infected wild-type plants.

Gene ID	TAIR Functional description	log_2_FC
AT4G37150	Methyl esterase 9	−0.8
AT2G14610	Pathogenesis-related gene 1	−1.2
AT1G75040	Pathogenesis-related gene 5	−0.7
AT1G19250	Flavin-dependent monooxygenase 1	−1.0
AT3G04720	Pathogenesis-related 4	−0.6
AT4G12470	Azelaic acid induced 1	−0.6
AT5G36970	NDR1/HIN1-like 25	−1.33
AT1G02205	Fatty acid hydroxylase superfamily	−1.2

Annotations were manually curated. FC: fold change.

**Table 2:** Defence-related proteins whose abundance differs between infected GC and infected wild-type plants.

ID gene	TAIR Functional description	log_2_FC
AT3G54960	Disulfide isomerase-like	1.09
AT5G09650	Pyrophosphorylase 6	−1.10
AT1G80460	Non host resistance to *P. syringae* pv. *phaseolicola* 1	−1.21
AT3G04120	Glyceraldehyde-3-phosphate dehydrogenase c subunit	−1.23
AT1G76680	12-Oxophytodienoate reductase 1	−1.42
AT4G08900	Arginine amidohydrolase 1	−1.48
AT2G34040	Apoptosis inhibitory protein 5	−1.72
AT3G15730	Phospholipase D alpha 1	−1.81
AT3G26650	Glyceraldehyde 3-phosphate dehydrogenase a subunit	−1.81
AT1G22410	Class-II DAHP synthetase	−2.01
AT5G43940	S-Nitrosoglutathione reductase	−2.11
AT3G44300	Nitrilase 2	−2.44
AT4G35790	Phospholipase D delta	−2.47
AT4G37980	Cinnamyl-alcohol dehydrogenase 7	−2.84
AT1G04980	Protein disulfide isomerase 10	−3.16
AT3G53180	Nodulin/glutamine synthase-like protein	−3.55
AT2G20630	PP2C induced by AvrRPM1	−3.62
AT4G01070	UDP-glucose-dependent glucosyltransferase 72 B1	−4.18
AT1G52400	Beta-glucosidase 1	−4.18
AT4G12720	*Arabidopsis thaliana* Nudix hydrolase homolog 7 (Nudt7)	−4.94
AT3G60190	Dynamin-related protein 1E, also known as EDR3	−5.64
AT2G41560	Autoinhibited Ca^2+^-ATPase, isoform 4	−11.48

Annotations were manually curated. FC: fold change.
