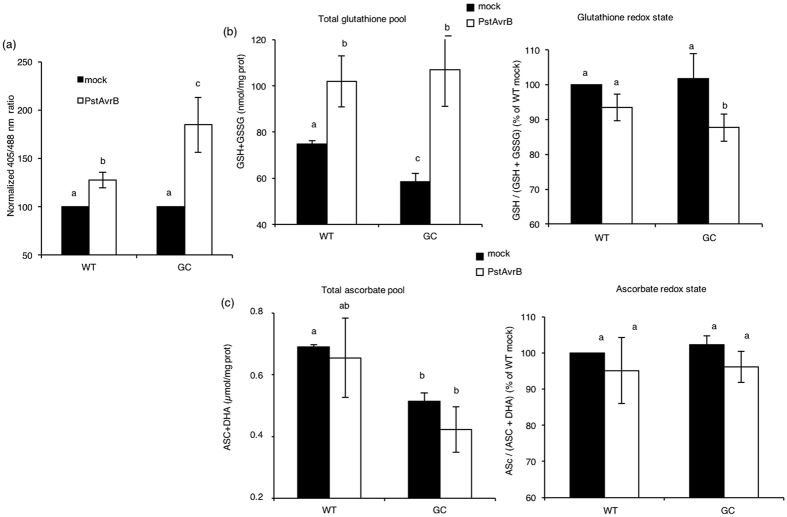# Corrigendum: Constitutive cyclic GMP accumulation in *Arabidopsis thaliana* compromises systemic acquired resistance induced by an avirulent pathogen by modulating local signals

**DOI:** 10.1038/srep46593

**Published:** 2017-05-03

**Authors:** Jamshaid Hussain, Jian Chen, Vittoria Locato, Wilma Sabetta, Smrutisanjita Behera, Sara Cimini, Francesca Griggio, Silvia Martínez-Jaime, Alexander Graf, Mabrouk Bouneb, Raman Pachaiappan, Paola Fincato, Emanuela Blanco, Alex Costa, Laura De Gara, Diana Bellin, Maria Concetta de Pinto, Elodie Vandelle

Scientific Reports 6: Article number: 36423; 10.1038/srep36423 published online: 11 04 2016; updated: 05 03 2017.

This Article contains an error in Figure 3c, where the y-axis ‘ASC + DHA (*μ*mol/mg prot)’ is incorrectly given as ‘ASC + DHA (nmol/mg prot)’. The correct Figure 3 appears below as Figure 1.

## Figures and Tables

**Figure 1:**