# Supplementary material for: Constitutive cyclic GMP accumulation in Arabidopsis thaliana compromises systemic acquired resistance induced by an avirulent pathogen by modulating local signals
Source: Sci Rep. 2016 Nov 4;6:36423. doi: 10.1038/srep36423 (PMC5095659; doi:10.1038/srep36423)
Supplement: Supplementary Information [file srep36423-s1.pdf]

Constitutive cyclic GMP accumulation in *Arabidopsis thaliana* compromises systemic acquired resistance induced by an avirulent pathogen by modulating local signals

Jamshaid Hussain[1¶a], Jian Chen[1¶b], Vittoria Locato[2¶], Wilma Sabetta[3¶c], Smrutisanjita Behera[4], Sara Cimini[2], Francesca Griggio[1], Silvia Martínez-Jaime[5], Alexander Graf[5], Mabrouk Bouneb[1,d], Raman Pachaiappan[1,e], Paola Fincato[2], Emanuela Blanco[3], Alex Costa[4], Laura De Gara[2], Diana Bellin[1,*], Maria Concetta de Pinto[6] and Elodie Vandelle[1,*]

[1]Department of Biotechnology, University of Verona, Verona, Italy

[2]Unit of Food Science and Nutrition, Department of Medicine, Università Campus Bio-Medico di Roma, Rome, Italy

[3]Institute of Biosciences and Bioresources – CNR, Research Division Bari, Bari, Italy

[4]Department of Biosciences, University of Milan, Milano, Italy

[5]Max Planck Institute of Molecular Plant Physiology, Potsdam-Golm, Germany

[6]Department of Biology, University of Bari "Aldo Moro", Bari, Italy

[*]Corresponding authors:

Elodie Vandelle, Dipartimento di Biotecnologie, Università degli Studi di Verona, Strada Le Grazie 15, 37134 Verona (Italy), Tel: +39 0458027826/Fax: +39 0458027929, Email: elodiegenevieve.vandelle@univr.it

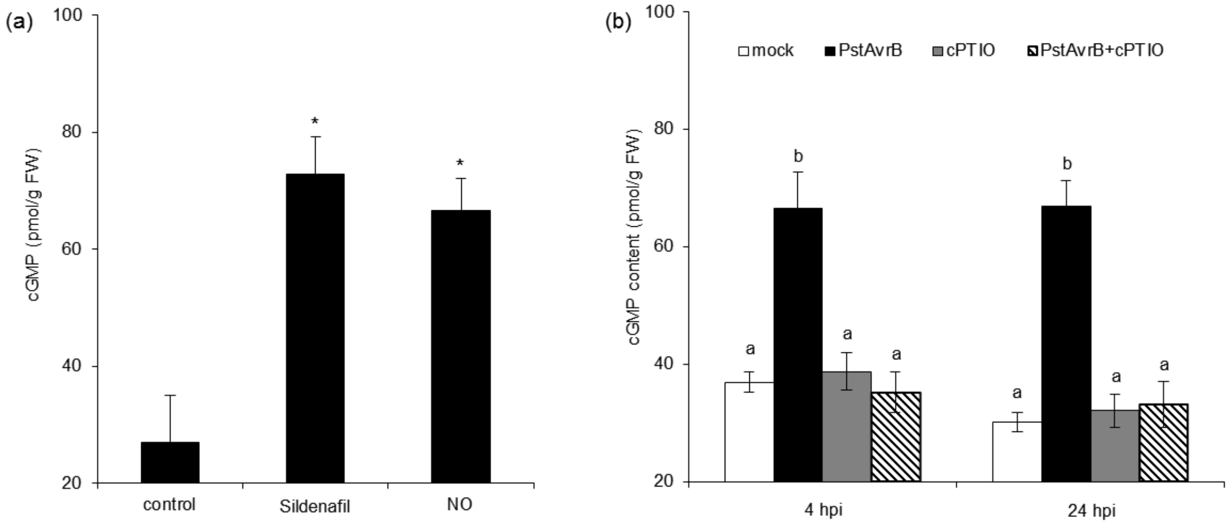

Supplementary Figure S1. Effect of phosphodiesterase inhibitor infiltration or NO fumigation on cGMP levels in *A. thaliana* and suppression of the *PstAvrB*-induced increase in cGMP level by the NO scavenger cPTIO. *A. thaliana* Col-0 leaves were infiltrated with sildenafil citrate (1 mg/mL) for 4 hours or fumigated with NO gas 100 ppm for 3 hours (a) or infected with *PstAvrB* (10^7 cfu/mL) for 4 and 24 hours in the presence or absence of cPTIO (500 µM) (b). In each experiment, mock-treated plants were used as controls. cGMP content was measured using AlphaScreen technology. Values are means ± SE from three independent experiments. Asterisks or different letters indicate a statistical difference ($p < 0.05$) according to Student's *t*-test (a) or ANOVA test (b). FW: fresh weight.

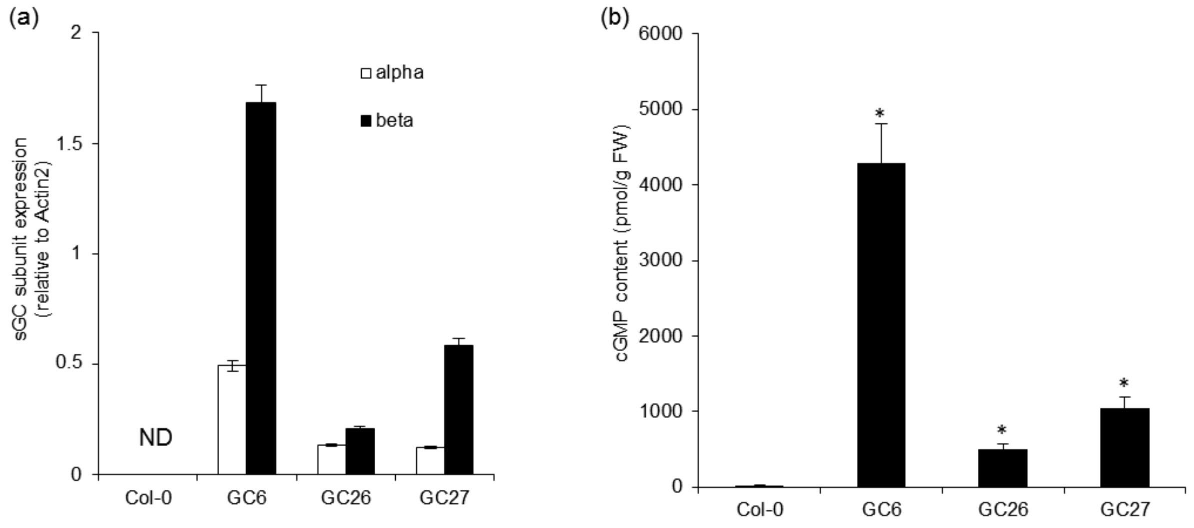

Supplementary Figure S2. Transgenic *A. thaliana* GC lines showing the expression of both the alpha and beta soluble guanylate cyclase subunits and displaying high constitutive levels of cGMP. (a) Total RNA was extracted from naïve *A. thaliana* Col-0 leaves and three independent homozygous GC lines. Real-time RT-PCR was carried out using subunit-specific primers. The expression level of genes encoding the alpha and beta subunits was normalized to the expression level of *Actin2*. (b) The cGMP content was measured in naïve *A. thaliana* Col-0 leaves and three independent GC lines using AlphaScreen technology. Values are means ± SE from two independent experiments. Asterisks indicate a statistical difference ($p < 0.05$) according to Student's *t*-test. FW: fresh weight.

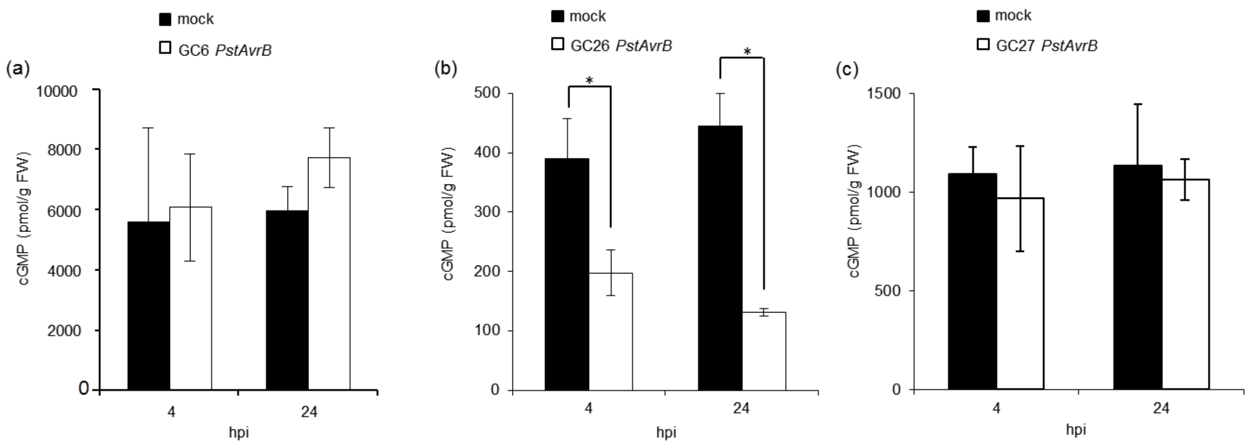

Supplementary Figure S3. High cGMP content is maintained in GC6 and GC27 lines at similar level during the pathogen infection but not in line GC26. The cGMP content was measured in three independent GC lines, namely line GC6 (a), GC26 (b) and GC27 (c) following infection with the avirulent bacterial pathogen *PstAvrB* (10^7 cfu/mL) at 4 and 24 hours post-infection. Values are means ± SE from two independent experiments. Asterisks indicate a statistical difference ($p < 0.05$) according to Student's *t*-test. FW: fresh weight.

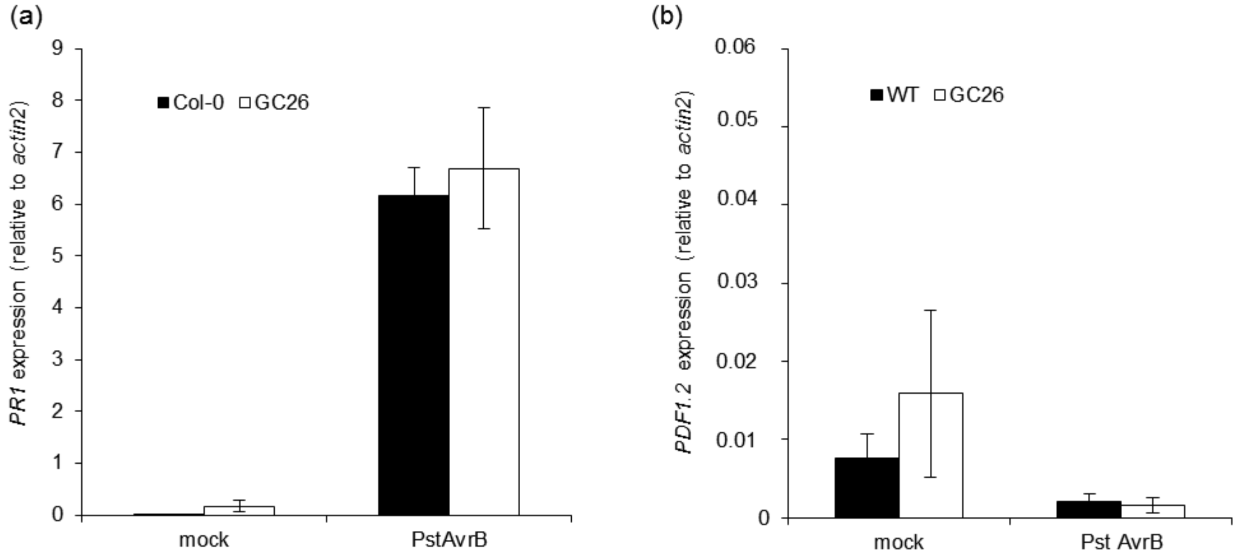

Supplementary Figure S4. Line GC26 displays normal defense gene expression in response to infection with *PstAvrB*. Leaf samples were collected from *A. thaliana* Col-0 leaves or transgenic GC26 line 12 hpi with the avirulent pathogen *PstAvrB* (10^7 cfu/mL) for the analysis of the expression of *PR-1*(a) and *PDF1.2* (b) by real-time RT-PCR using gene-specific primers. The expression level of each gene was normalized to that of *Actin2*. In each experiment, mock-infiltrated plants were used as controls. Values are means ± SEM of at least three biological replicates.

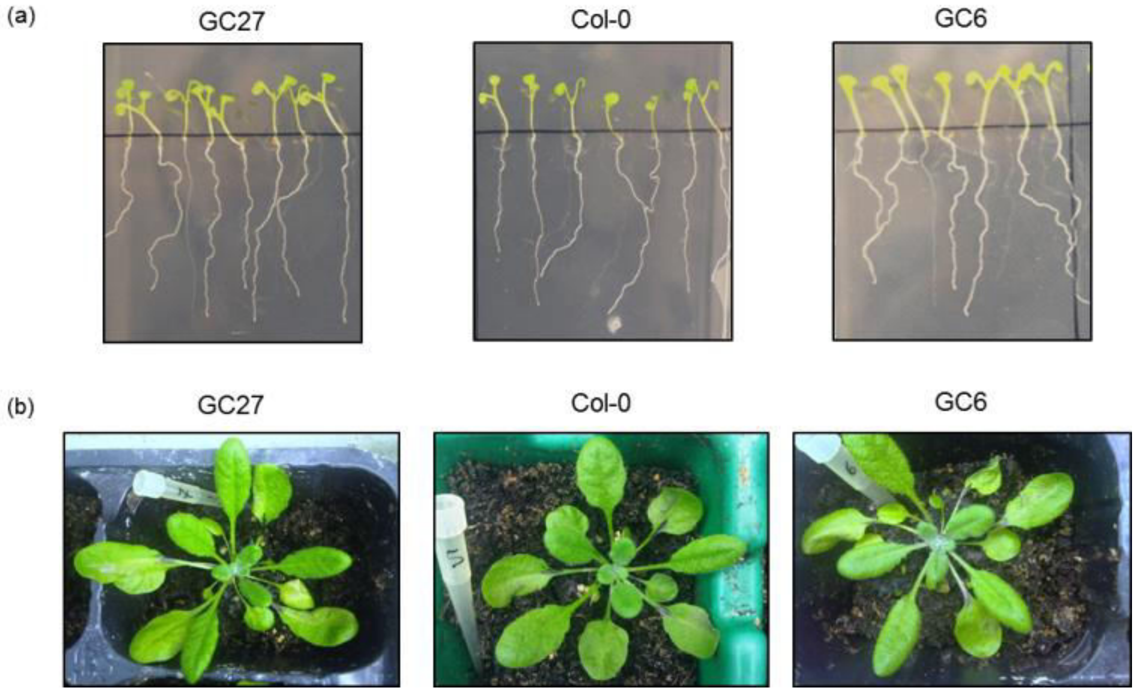

Supplementary Figure S5. Transgenic GC27 and GC6 lines do not show growth phenotype alteration. (a) Seeds of *Arabidopsis thaliana* Col-0, GC6 and GC27 lines were sterilized and sown on MS agar. Pictures were taken 7 days after germination. (b) Seeds of *Arabidopsis thaliana* Col-0, GC6 and GC27 lines were sown on soil. Pictures represent 5-week old plans 1 day after infiltration with *PstAvrB* (10^7 cfu/mL). Infiltrated leaves are indicated with a black dot.

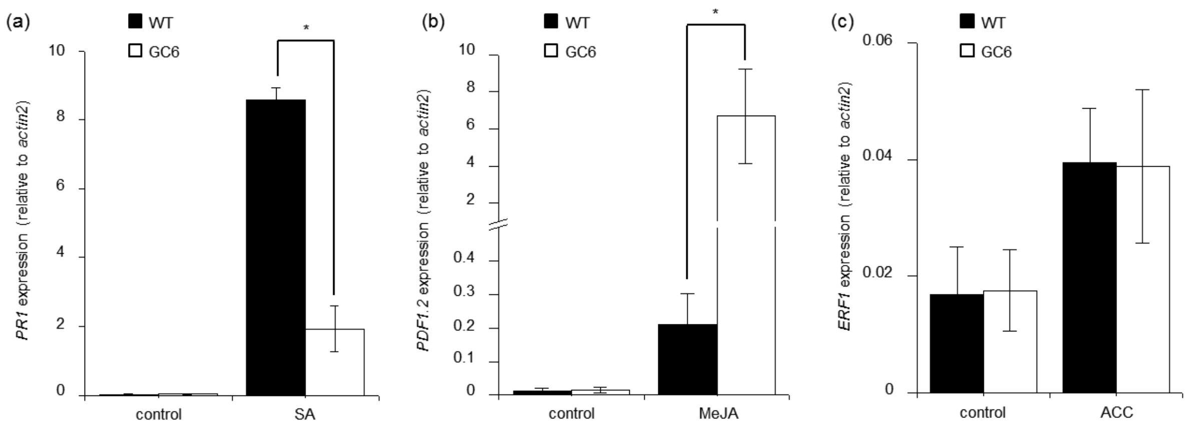

Supplementary Figure S6. High cGMP level deregulates hormone-dependent defense gene expression downstream of jasmonate and salicylic acid. Leaf samples were collected from *A. thaliana* Col-0 leaves sprayed with SA (1 mM), MeJA (50µM) or ACC (1 mM) for the analysis of the expression of *PR1* (a)*, PDF1.2* (b) or *ERF1* (c), respectively, by real-time RT-PCR using gene-specific primers. The expression level of each gene was normalized to that of *Actin2*. In each experiment, mock-sprayed plants were used as controls. Values are means ± SEM of at least three biological replicates. Asterisks indicate a statistical difference ($p < 0.05$) according to Student's *t*-test. SA, salicylic acid; MeJA, methyl jasmonate; ACC, 1-aminocyclopropane-1-carboxylic acid.

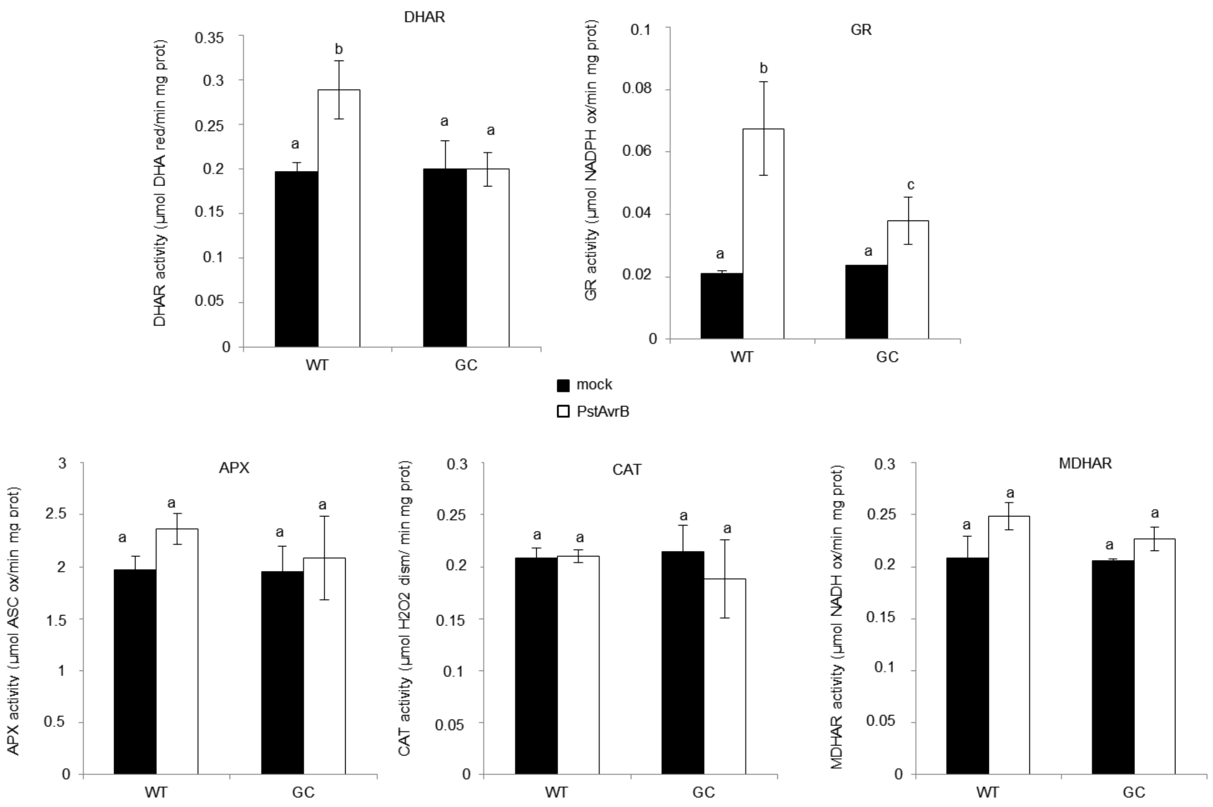

Supplementary Figure S7. Ascorbate-glutathione redox enzyme activities in wild-type and transgenic GC plants infected with avirulent *PstAvrB*. *A. thaliana* wild-type Col-0 and transgenic GC plants were infected with *PstAvrB* (10^7 cfu/mL) and leaf samples were collected 24 hpi. Enzyme activities were measured in total protein extracts. In each experiment, mock-infiltrated plants were used as controls. Values are means ± SE of three independent experiments. Different letters indicate a statistical difference ($p < 0.05$) based on ANOVA. DHAR, dihydroascorbate reductase; GR, glutathione reductase; APX, ascorbate peroxidase; CAT, catalase; MDHAR, monodihydroascorbate reductase.

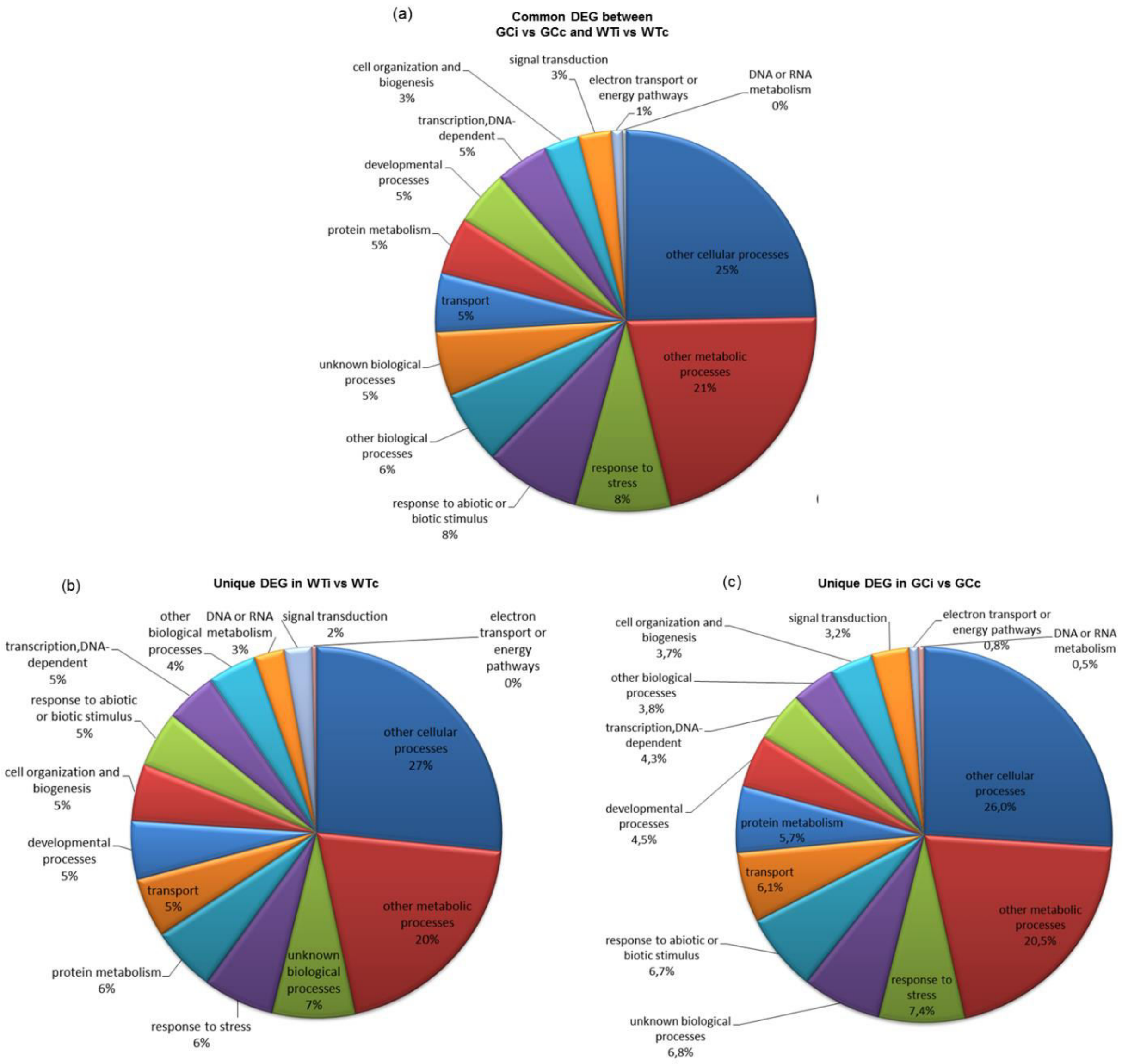

Supplementary Figure S8. Distribution of functional categories of differentially-expressed genes (DEGs). (a) Common DEGs in infected GC and infected WT plants. (b) Unique DEGs in infected WT plants. (c) Unique DEGs in infected GC plants. DEGs are genes whose expression was modulated with log 2 ratio $\geq +1$ or ≤ -1, $p \leq 0.05$) following infection with *PstAvrB* (10^7 cfu/mL). Functional annotation was carried out using TAIR.

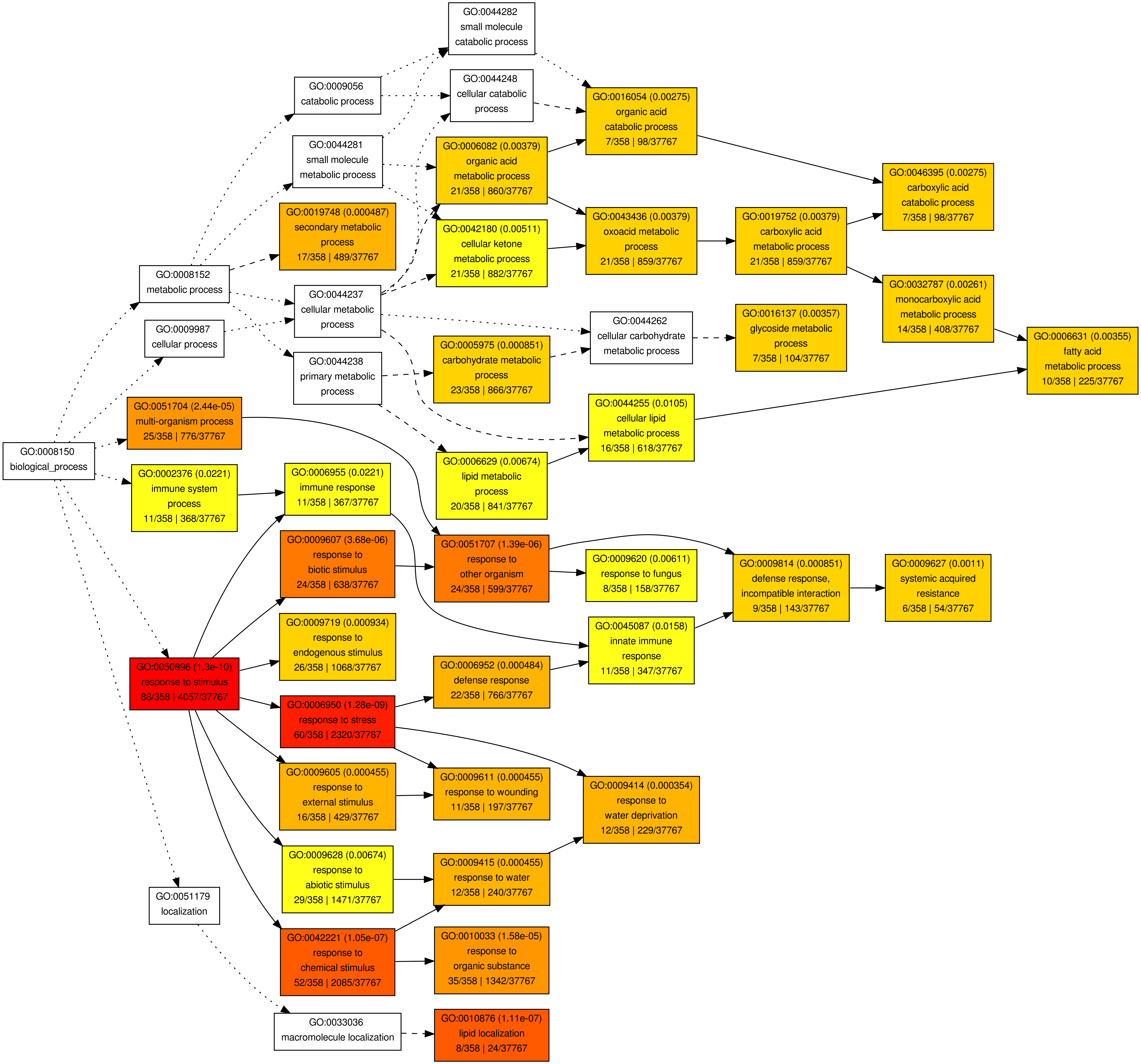
GO:0044282
small molecule
catabolic process
GO:0009056
catabolic process
GO:0044248
cellular catabolic
process
GO:0016054 (0.00275)
organic acid
catabolic process
7/358 | 98/37767
GO:0044281
small molecule
metabolic process
GO:0006082 (0.00379)
organic acid
metabolic process
21/358 | 860/37767
GO:0046395 (0.00275)
carboxylic acid
catabolic process
7/358 | 98/37767
GO:0019748 (0.000487)
secondary metabolic
process
17/358 | 489/37767
GO:0042180 (0.00511)
cellular ketone
metabolic process
21/358 | 882/37767
GO:0043436 (0.00379)
oxoacid metabolic
process
21/358 | 859/37767
GO:0019752 (0.00379)
carboxylic acid
metabolic process
21/358 | 859/37767
GO:0008152
metabolic process
GO:0044237
cellular metabolic
process
GO:0032787 (0.00261)
monocarboxylic acid
metabolic process
14/358 | 408/37767
GO:0009987
cellular process
GO:0044262
cellular carbohydrate
metabolic process
GO:0016137 (0.00357)
glycoside metabolic
process
7/358 | 104/37767
GO:0006631 (0.00355)
fatty acid
metabolic process
10/358 | 225/37767
GO:0044238
primary metabolic
process
GO:0005975 (0.000851)
carbohydrate metabolic
process
23/358 | 866/37767
GO:0044255 (0.0105)
cellular lipid
metabolic process
16/358 | 618/37767
GO:0051704 (2.44e-05)
multi-organism process
25/358 | 776/37767
GO:0008150
biological_process
GO:0006629 (0.00674)
lipid metabolic
process
20/358 | 841/37767
GO:0002376 (0.0221)
immune system
process
11/358 | 368/37767
GO:0006955 (0.0221)
immune response
11/358 | 367/37767
GO:0009607 (3.68e-06)
response to
biotic stimulus
24/358 | 638/37767
GO:0051707 (1.39e-06)
response to
other organism
24/358 | 599/37767
GO:0009620 (0.00611)
response to fungus
8/358 | 158/37767
GO:0009814 (0.000851)
defense response,
incompatible interaction
9/358 | 143/37767
GO:0009627 (0.0011)
systemic acquired
resistance
6/358 | 54/37767
GO:0009719 (0.000934)
response to
endogenous stimulus
26/358 | 1068/37767
GO:0045087 (0.0158)
innate immune
response
11/358 | 347/37767
GO:0050896 (1.3e-10)
response to stimulus
88/358 | 4057/37767
GO:0006952 (0.000484)
defense response
22/358 | 766/37767
GO:0006950 (1.28e-09)
response to stress
60/358 | 2320/37767
GO:0009605 (0.000455)
response to
external stimulus
16/358 | 429/37767
GO:0009611 (0.000455)
response to wounding
11/358 | 197/37767
GO:0009414 (0.000354)
response to
water deprivation
12/358 | 229/37767
GO:0009628 (0.00674)
response to
abiotic stimulus
29/358 | 1471/37767
GO:0009415 (0.000455)
response to water
12/358 | 240/37767
GO:0051179
localization
GO:0042221 (1.05e-07)
response to
chemical stimulus
52/358 | 2085/37767
GO:0010033 (1.58e-05)
response to
organic substance
35/358 | 1342/37767
GO:0033036
macromolecule localization
GO:0010876 (1.11e-07)
lipid localization
8/358 | 24/37767

Supplementary Figure S9. Graphical presentation of over-represented functional categories relative to the whole genome by Gene Ontology (GO) annotation of downregulated genes in GC plants infected with *PstAvrB* (10^7 cfu/mL) compared to infected wild-type plants. The analysis was carried out using AgriGO (http://bioinfo.cau.edu.cn/agriGO/analysis.php).

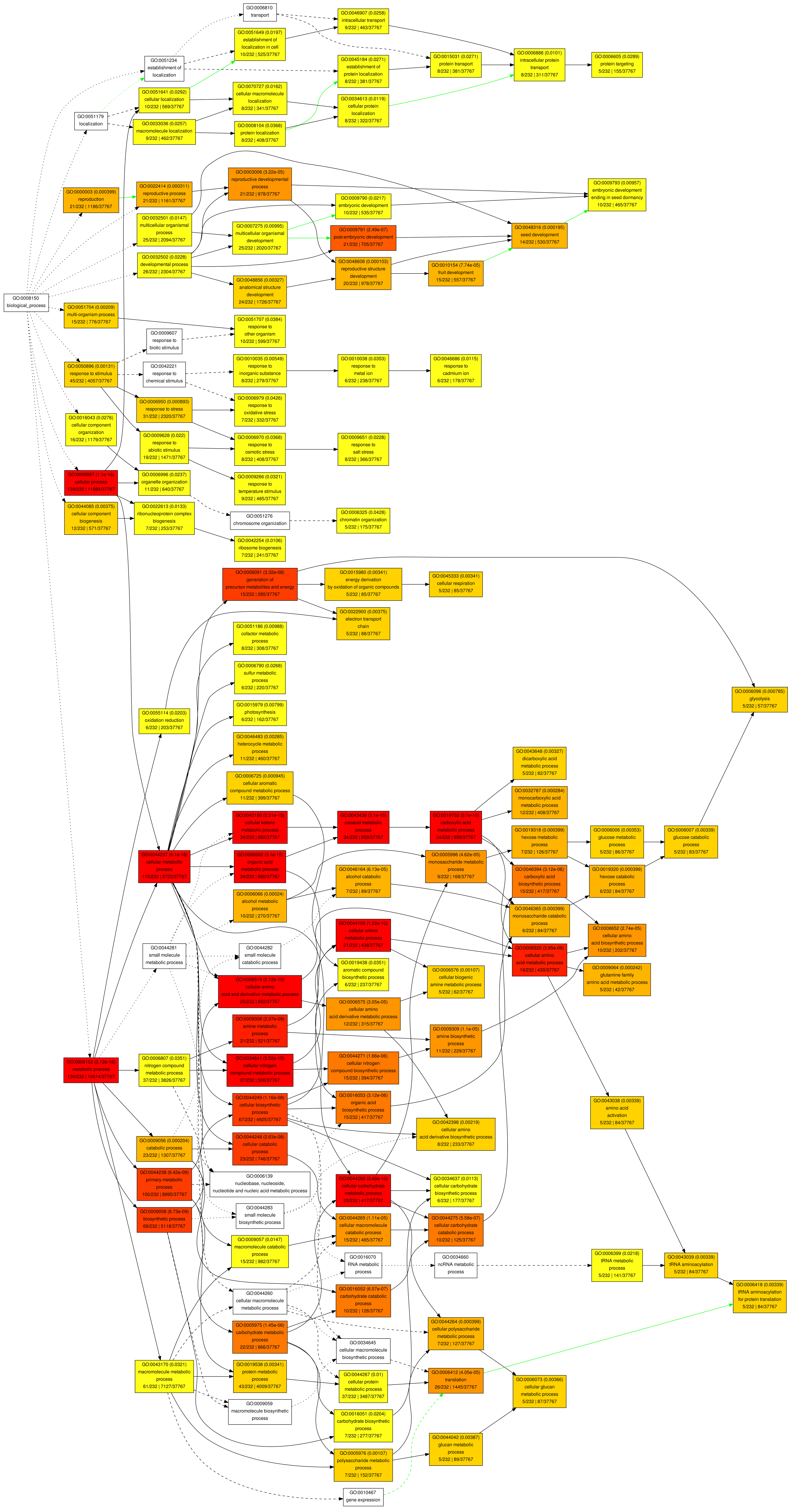

GO:0008150 biological_process
GO:0006810 transport
GO:0051234 establishment of localization
GO:0051179 localization
GO:0046907 (0.0258) intracellular transport 9/232 | 463/37767
GO:0051649 (0.0197) establishment of localization in cell 10/232 | 525/37767
GO:0045184 (0.0271) establishment of protein localization 8/232 | 381/37767
GO:0015031 (0.0271) protein transport 8/232 | 381/37767
GO:0006886 (0.0101) intracellular protein transport 8/232 | 311/37767
GO:0006605 (0.0289) protein targeting 5/232 | 155/37767
GO:0051641 (0.0292) cellular localization 10/232 | 569/37767
GO:0070727 (0.0162) cellular macromolecule localization 8/232 | 341/37767
GO:0034613 (0.0119) cellular protein localization 8/232 | 322/37767
GO:0033036 (0.0257) macromolecule localization 9/232 | 462/37767
GO:0008104 (0.0368) protein localization 8/232 | 408/37767
GO:0000003 (0.000399) reproduction 21/232 | 1186/37767
GO:0022414 (0.000311) reproductive process 21/232 | 1161/37767
GO:0003006 (3.22e-05) reproductive developmental process 21/232 | 978/37767
GO:0009793 (0.00957) embryonic development ending in seed dormancy 10/232 | 465/37767
GO:0032501 (0.0147) multicellular organismal process 25/232 | 2094/37767
GO:0009790 (0.0217) embryonic development 10/232 | 535/37767
GO:0007275 (0.00995) multicellular organismal development 25/232 | 2020/37767
GO:0009791 (2.49e-07) post-embryonic development 21/232 | 705/37767
GO:0048316 (0.000195) seed development 14/232 | 530/37767
GO:0032502 (0.0228) developmental process 26/232 | 2304/37767
GO:0048608 (0.000103) reproductive structure development 20/232 | 978/37767
GO:0010154 (7.74e-05) fruit development 15/232 | 557/37767
GO:0048856 (0.00327) anatomical structure development 24/232 | 1726/37767
GO:0051704 (0.00209) multi-organism process 15/232 | 776/37767
GO:0051707 (0.0384) response to other organism 10/232 | 599/37767
GO:0009607 response to biotic stimulus
GO:0050896 (0.00131) response to stimulus 45/232 | 4057/37767
GO:0042221 response to chemical stimulus
GO:0010035 (0.00549) response to inorganic substance 8/232 | 279/37767
GO:0010038 (0.0353) response to metal ion 6/232 | 238/37767
GO:0046686 (0.0115) response to cadmium ion 6/232 | 178/37767
GO:0006950 (0.000893) response to stress 31/232 | 2320/37767
GO:0006979 (0.0426) response to oxidative stress 7/232 | 332/37767
GO:0016043 (0.0276) cellular component organization 16/232 | 1179/37767
GO:0009628 (0.022) response to abiotic stimulus 19/232 | 1471/37767
GO:0006970 (0.0368) response to osmotic stress 8/232 | 408/37767
GO:0009651 (0.0228) response to salt stress 8/232 | 366/37767
GO:0009987 (1.1e-15) cellular process 135/232 | 11604/37767
GO:0006996 (0.0237) organelle organization 11/232 | 640/37767
GO:0009266 (0.0321) response to temperature stimulus 9/232 | 485/37767
GO:0044085 (0.00375) cellular component biogenesis 12/232 | 571/37767
GO:0022613 (0.0133) ribonucleoprotein complex biogenesis 7/232 | 253/37767
GO:0051276 chromosome organization
GO:0006325 (0.0428) chromatin organization 5/232 | 175/37767
GO:0042254 (0.0106) ribosome biogenesis 7/232 | 241/37767
GO:0006091 (3.32e-08) generation of precursor metabolites and energy 15/232 | 285/37767
GO:0015980 (0.00341) energy derivation by oxidation of organic compounds 5/232 | 85/37767
GO:0045333 (0.00341) cellular respiration 5/232 | 85/37767
GO:0022900 (0.00375) electron transport chain 5/232 | 88/37767
GO:0051186 (0.00988) cofactor metabolic process 8/232 | 308/37767
GO:0006790 (0.0268) sulfur metabolic process 6/232 | 220/37767
GO:0006096 (0.000785) glycolysis 5/232 | 57/37767
GO:0055114 (0.0203) oxidation reduction 6/232 | 203/37767
GO:0015979 (0.00799) photosynthesis 6/232 | 162/37767
GO:0046483 (0.00285) heterocycle metabolic process 11/232 | 460/37767
GO:0043648 (0.00327) dicarboxylic acid metabolic process 5/232 | 82/37767
GO:0006725 (0.000945) cellular aromatic compound metabolic process 11/232 | 399/37767
GO:0032787 (0.000284) monocarboxylic acid metabolic process 12/232 | 408/37767
GO:0042180 (5.51e-15) cellular ketone metabolic process 34/232 | 882/37767
GO:0043436 (3.1e-15) oxoacid metabolic process 34/232 | 859/37767
GO:0019752 (3.1e-15) carboxylic acid metabolic process 34/232 | 859/37767
GO:0019318 (0.000399) hexose metabolic process 7/232 | 126/37767
GO:0006006 (0.00353) glucose metabolic process 5/232 | 86/37767
GO:0006007 (0.00339) glucose catabolic process 5/232 | 83/37767
GO:0044237 (6.1e-16) cellular metabolic process 115/232 | 9722/37767
GO:0006082 (3.1e-15) organic acid metabolic process 34/232 | 860/37767
GO:0005996 (4.62e-05) monosaccharide metabolic process 9/232 | 168/37767
GO:0046394 (3.12e-06) carboxylic acid biosynthetic process 15/232 | 417/37767
GO:0019320 (0.000399) hexose catabolic process 6/232 | 84/37767
GO:0046164 (6.13e-05) alcohol catabolic process 7/232 | 89/37767
GO:0006066 (0.00024) alcohol metabolic process 10/232 | 270/37767
GO:0046365 (0.000399) monosaccharide catabolic process 6/232 | 84/37767
GO:0044106 (1.22e-10) cellular amine metabolic process 21/232 | 438/37767
GO:0008652 (2.74e-05) cellular amino acid biosynthetic process 10/232 | 202/37767
GO:0044281 small molecule metabolic process
GO:0044282 small molecule catabolic process
GO:0006520 (3.95e-09) cellular amino acid metabolic process 19/232 | 430/37767
GO:0019438 (0.0351) aromatic compound biosynthetic process 6/232 | 237/37767
GO:0006576 (0.00107) cellular biogenic amine metabolic process 5/232 | 62/37767
GO:0009064 (0.000242) glutamine family amino acid metabolic process 5/232 | 42/37767
GO:0006519 (2.12e-10) cellular amino acid and derivative metabolic process 25/232 | 582/37767
GO:0006575 (3.05e-05) cellular amino acid derivative metabolic process 12/232 | 315/37767
GO:0009308 (2.07e-09) amine metabolic process 21/232 | 521/37767
GO:0009309 (1.1e-05) amine biosynthetic process 11/232 | 229/37767
GO:0044271 (1.66e-06) cellular nitrogen compound biosynthetic process 15/232 | 394/37767
GO:0008152 (3.12e-16) metabolic process 130/232 | 10614/37767
GO:0006807 (0.0351) nitrogen compound metabolic process 37/232 | 3826/37767
GO:0034641 (5.52e-15) cellular nitrogen compound metabolic process 27/232 | 506/37767
GO:0043038 (0.00339) amino acid activation 5/232 | 84/37767
GO:0044249 (1.16e-08) cellular biosynthetic process 67/232 | 4925/37767
GO:0016053 (3.12e-06) organic acid biosynthetic process 15/232 | 417/37767
GO:0042398 (0.00219) cellular amino acid derivative biosynthetic process 8/232 | 233/37767
GO:0009056 (0.000204) catabolic process 23/232 | 1307/37767
GO:0044248 (2.63e-08) cellular catabolic process 23/232 | 746/37767
GO:0044238 (6.42e-09) primary metabolic process 100/232 | 8995/37767
GO:0006139 nucleobase, nucleoside, nucleotide and nucleic acid metabolic process
GO:0044262 (3.49e-10) cellular carbohydrate metabolic process 20/232 | 417/37767
GO:0034637 (0.0113) cellular carbohydrate biosynthetic process 6/232 | 177/37767
GO:0009058 (8.73e-09) biosynthetic process 69/232 | 5118/37767
GO:0044283 small molecule biosynthetic process
GO:0044265 (1.11e-05) cellular macromolecule catabolic process 15/232 | 465/37767
GO:0044275 (5.58e-07) cellular carbohydrate catabolic process 10/232 | 125/37767
GO:0009057 (0.0147) macromolecule catabolic process 15/232 | 982/37767
GO:0016070 RNA metabolic process
GO:0034660 ncRNA metabolic process
GO:0006399 (0.0218) tRNA metabolic process 5/232 | 141/37767
GO:0043039 (0.00339) tRNA aminoacylation 5/232 | 84/37767
GO:0006418 (0.00339) tRNA aminoacylation for protein translation 5/232 | 84/37767
GO:0044260 cellular macromolecule metabolic process
GO:0016052 (6.57e-07) carbohydrate catabolic process 10/232 | 128/37767
GO:0044264 (0.000399) cellular polysaccharide metabolic process 7/232 | 127/37767
GO:0005975 (1.45e-06) carbohydrate metabolic process 22/232 | 866/37767
GO:0034645 cellular macromolecule biosynthetic process
GO:0006412 (4.05e-05) translation 26/232 | 1445/37767
GO:0043170 (0.0321) macromolecule metabolic process 61/232 | 7127/37767
GO:0019538 (0.00341) protein metabolic process 43/232 | 4009/37767
GO:0044267 (0.01) cellular protein metabolic process 37/232 | 3487/37767
GO:0006073 (0.00366) cellular glucan metabolic process 5/232 | 87/37767
GO:0009059 macromolecule biosynthetic process
GO:0016051 (0.0204) carbohydrate biosynthetic process 7/232 | 277/37767
GO:0044042 (0.00387) glucan metabolic process 5/232 | 89/37767
GO:0005976 (0.00107) polysaccharide metabolic process 7/232 | 152/37767
GO:0010467 gene expression

Figure S10. Graphical presentation of over-represented functional categories relative to the whole genome by Gene Ontology (GO) annotation of downregulated proteins in GC plants infected with *PstAvrB* (10^7 cfu/mL) compared to infected wild-type plants. The analysis was carried out using AgriGO (http://bioinfo.cau.edu.cn/agriGO/analysis.php).

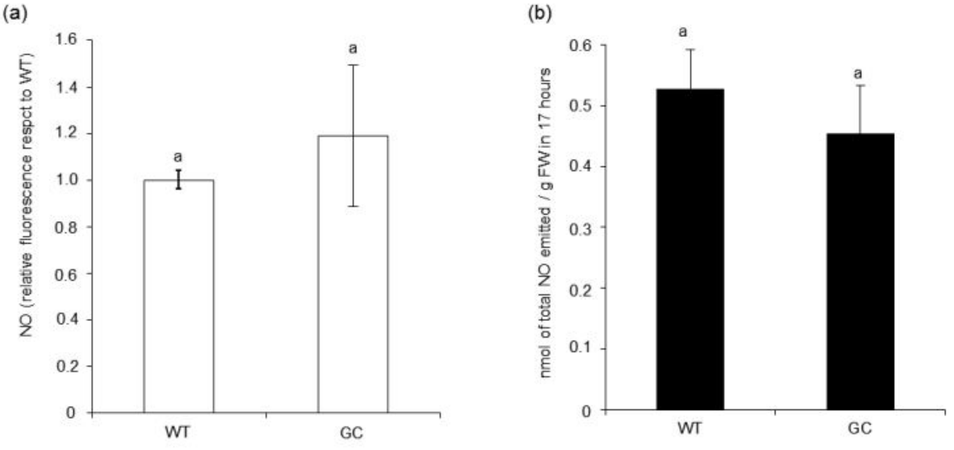

Supplementary Figure S11. Transgenic *A. thaliana* GC lines show the same NO accumulation at basal level and in response to infection with *PstAvrB*. (a) Entire detached leaves of both GC and wild-type genotypes were homogenized in 50 mM phosphate buffer, pH 7 in 1:4 (w:v) ratio. The extracts were centrifuged at 20,000 g for 15 min at 4°C. The supernatants were recovered and incubated with the fluorescent probe 4-Amino-5-methylamino-2′,7′-difluorescein diacetate (DAF-FM DA; 20 μM) for 30 min in the dark. The fluorescence intensity was measured at λexc=495 nm and λem 515 nm. (b) Gas-phase NO emission amount of *A. thaliana* Col-0 and GC plants in response to infection with *PstAvrB* revealed by chemiluminescence according to Chen et al. (2014). Leaves were infiltrated with avirulent bacterial pathogen and placed in the nutrient solution. NO emission was measured by chemiluminescence and recorded for 17 hours. Total NO emitted during the course of the HR was determined by calculating the whole area of chemiluminescence signal. FW, fresh weight. The same letter indicates no statistical difference ($p < 0.05$) according to ANOVA test.

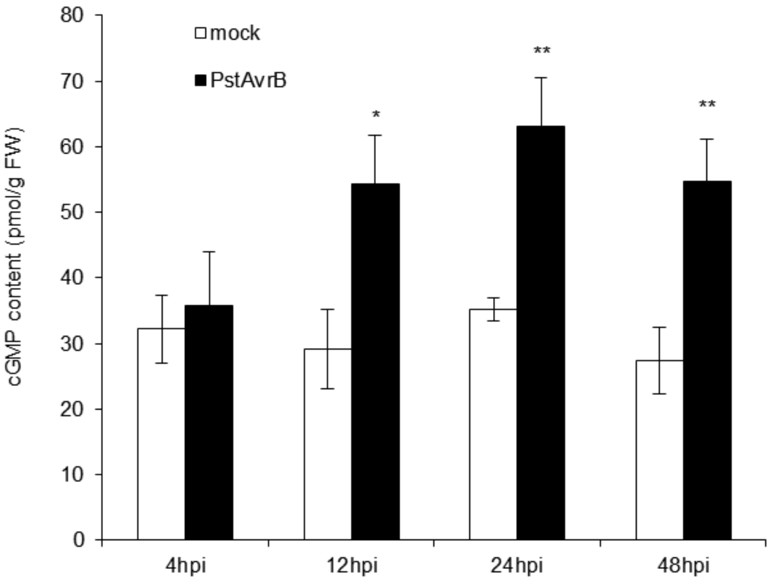

Supplementary Figure S12. Distal leaves of *Arabidopsis thaliana* plants show an accumulation of cGMP following infection of primary leaves with the avirulent pathogen *PstAvrB*. Measurement of the cGMP content in *A. thaliana* Col-0 systemic leaves following primary infection of local leaves with *PstAvrB* (10^7 cfu/mL) and collected at different time points during the course of infection. Values are means ± SE of three biological replicates, each including three technical replicates. Asterisks indicate a statistical difference ($p < 0.05$) according to Student's *t*-test. FW: fresh weight.

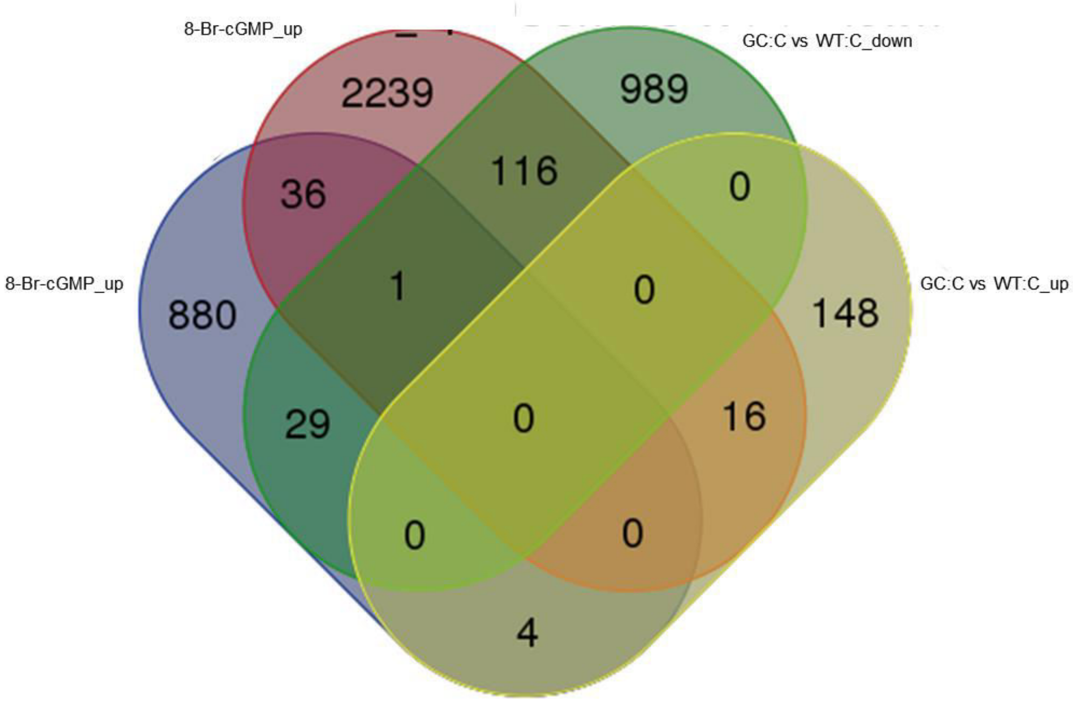

Supplementary Figure S13. Venn diagrams illustrating transcriptomic changes in *Arabidopsis thaliana* roots treated with 8-Br-cGMP (Maathuis, 2006) and transgenic *A. thaliana* sGC plants (reported herein). The diagrams were generated using VENNY (http://bioinfogp.cnb.csic.es/tools/venny/index.html).

Supplementary Table S1. List of primers used for gene expression analysis by real-time in this study. Primers were designed using the software *Primer3*.

Primers	Sequences
Actin2 forward	CTCATGCCATCCTCCGTCTT
Actin2 reverse	CAATTTCCCGCTCTGCTGTT
GC alpha forward	GGGTTATGGACCTCAAAGGT
GC alpha reverse	TGAATCGGGATGTCAGACAG
GC beta forward	AAGAGGCCTGTACCTGAGTG
GC beta reverse	ACAGATGGAGGGAGAACAGA
PR-1 forward	GCAACTGCAGACTCATACAC
PR-1 reverse	GTTGTAGTTAGCCTTCTCGC
PDF1.2 forward	CACCCTTATCTTCGCTGCTCTT
PDF1.2 reverse	TACACTTGTGTGCTGGGAAGAC
PR-5 forward	TCCTTGACCGGCGAGAGTT
PR-5 reverse	AGGAACAATTGCCCTACCACC
ERF1 forward	GAGGAAACACTCGATGAGACG
ERF1 reverse	GGAGCGGTGATCAAAGTCAC

Supplementary Table S2. Differentially expressed genes related to lipid metabolism/transport in infected GC plants compared to infected WT plants.

ID gene	TAIR Functional description	log_2FC
AT3G03480	Acetyl CoA:(Z)-3-hexen-1-ol acetyltransferase	-1,15
AT1G04580	Aldehyde oxidase 4	-1,96
AT4G39955	Alpha/beta-Hydrolases superfamily protein	-0,51
AT1G68620	Alpha/beta-Hydrolases superfamily protein	-0,77
AT2G39410	Alpha/beta-Hydrolases superfamily protein	-1,24
AT5G65390	Arabinogalactan protein 7	-1,98
AT4G12470	Azelaic acid induced 1	-0,62
AT4G25700	Beta-hydroxylase 1	-0,63
AT2G48130	Bifunctional inhibitor/lipid-transfer protein/seed storage 2S albumin superfamily protein	-3,54
AT5G16080	Carboxyesterase 17	-1,20
AT4G32810	Carotenoid cleavage dioxygenase 8	-2,14
AT1G01600	Cytochrome P45, family 86, subfamily A, polypeptide 4	-1,28
AT1G02205	Fatty acid hydroxylase superfamily	-1,15
AT5G45950	GDSL-like Lipase/Acylhydrolase superfamily protein	-0,64
AT3G50400	GDSL-like Lipase/Acylhydrolase superfamily protein	-1,98
AT1G06520	Glycerol-3-phosphate acyltransferase 1	-0,83
AT3G11430	Glycerol-3-phosphate acyltransferase 5	-1,85
AT2G41250	Haloacid dehalogenase-like hydrolase (HAD) superfamily protein	-1,15
AT2G38540	Lipid transfer protein 1	-0,58
AT2G38530	Lipid transfer protein 2	-0,86
AT5G59320	Lipid transfer protein 3	-0,60
AT5G59310	Lipid transfer protein 4	-1,10

AT5G13900	Lipid transfer-like protein VAS	-2,76
AT5G14180	Myzus persicae-induced lipase 1	-1,14
AT3G01420	Peroxidase superfamily protein (DOX1)	-3,04
AT2G34980	Phosphatidylinositolglycan synthase family protein	-1,13
AT5G05440	Polyketide cyclase/dehydrase and lipid transport superfamily protein	-0,72
AT1G02470	Polyketide cyclase/dehydrase and lipid transport superfamily protein	-0,79
AT5G21170	SNF1-related protein kinase regulatory subunit beta-1	-0,51
AT1G12090	Extensin-like protein	0,58
AT2G15050	Lipid transfer protein (LTP7), predicted PR protein	0,69
AT3G16370	GDSL-like Lipase/Acylhydrolase superfamily protein	0,53
AT3G47750	ATP binding cassette subfamily A4	0,86

Supplementary Table S3. Differentially expressed genes related to hormone metabolism/response in infected GC plants compared to infected WT plants.

ID gene	TAIR Functional description	log2FC
Salicylic acid		
AT4G16690	Methyl esterase 16	-0,71
AT4G37150	Methyl esterase 9	-0,83
AT3G32980	Peroxidase 32	-1,03
AT1G48000	Myb domain protein 112	-0,99
AT5G54230	Myb domain protein 49	-1,05
Jasmonic acid		
AT3G55970	Jasmonate-regulated gene 21	-0,59
AT1G30135	Jasmonate-zim-domain protein 8	-0,59
AT4G35770	Rhodanese/Cell cycle control phosphatase superfamily protein	-0,77
AT3G15500	NAC domain containing protein 3	-0,79
AT3G06490	Myb domain protein 18	-0,94
AT1G52410	TSK-associating protein 1	0,62
AT1G56650	Production of anthocyanin pigment 1	0,69
AT2G24210	Terpene synthase 1	0,79
AT4G10390	Protein kinase superfamily protein	0,64
AT5G15310	Myb domain protein 16	0,63
AT5G44420	Plant defensin 1.2	1,97
Ethylene		
AT1G43160	Ethylene-responsive transcription factor RAP2-6	-0,54
AT5G13330	Ethylene-responsive transcription factor ERF113	-0,55

AT3G04720	Pathogenesis-related 4	-0,62
AT1G21910	Ethylene-responsive transcription factor ERF012	-0,62
AT3G16770	Ethylene-responsive transcription factor RAP2-3	-0,66
AT1G71520	Ethylene-responsive transcription factor ERF020	-0,77
AT3G60490	Integrase-type DNA-binding superfamily protein	1,49
AT5G04950	Nicotianamine synthase 1	0,74
AT5G11590	Integrase-type DNA-binding superfamily protein	0,96
AT5G15310	Myb domain protein 16	0,63
AT5G44420	Plant defensin 1.2	1,97
Gibberellin		
AT2G36830	Gamma tonoplast intrinsic protein	-0,64
AT1G69530	Expansin A1	-0,68
AT1G26960	Homeobox protein 23	0,73
Abscisic acid		
AT1G20440	Dehydrin COR47	-0,53
AT4G34138	UDP-glucosyl transferase 73B1	-0,59
AT5G59320	Lipid transfer protein 3	-0,60
AT5G15960	Stress-responsive protein (KIN1) / stress-induced protein (KIN1)	-0,78
AT5G59310	Lipid transfer protein 4	-1,10
AT2G29090	Cytochrome P45, family 77, subfamily A, polypeptide 2	-1,31
AT4G28110	Myb domain protein 41	-1,44
AT1G04580	Aldehyde oxidase 4	-1,96
AT1G52400	Beta glucosidase 18	0,68
ATCG00490	Ribulose-bisphosphate carboxylases	2,52
Auxin		
AT1G21410	F-box/RNI-like superfamily protein	-0,53

AT2G23170	Auxin-responsive GH3 family protein	-0,55
AT1G19850	Auxin response factor 5	-0,63
AT1G28130	Auxin-responsive GH3 family protein	-0,72
AT3G48360	BTB and TAZ domain protein 2	-0,75
AT4G13790	SAUR-like auxin-responsive protein family	-0,76
AT1G75580	SAUR-like auxin-responsive protein family	-1,07
AT3G44300	Nitrilase 2	-1,63
AT4G32810	Carotenoid cleavage dioxygenase 8	-2,14
AT1G12090	Extensin-like protein	0,58
AT4G34760	SAUR-like auxin-responsive protein family	0,73
AT5G64770	Root meristem growth factor 9	0,75
Cytokinin		
AT2G36800	Cytokinin-O-glucosyltransferase 3	-0,88
AT1G26210	SOB five-like 1	0,89
AT1G78370	Glutathione S-transferase TAU 2	0,74
AT3G16360	HPT phosphotransmitter 4	1,64
AT5G03760	Nucleotide-diphospho-sugar transferases superfamily protein	0,79
ATCG00130	ATPase, F complex, subunit B/B', bacterial/chloroplast	1,25

Supplementary Table S4. Proteins accumulating differentially in GC plants compared to WT plants at basal level.

**Gene ID**	**Gene name**	**log2FC**
AT3G27690	LHCB2	2,876619
AT1G76140	Prolyl oligopeptidase	-1,00197
AT4G32940	Gamma vacuolar processing enzyme	-1,59215
AT5G52310	Cold regulated 78	-1,13036
AT1G09010	Glycoside hydrolase family 2 protein	-1,24632
AT3G62750	Beta glucosidase 8	-2,0239
AT3G15730	Phospholipase D alpha 1	-1,25744
AT4G21960	PRXR1	-1,30214
AT4G34230	Cinnamyl alcohol dehydrogenase 5	-1,4196
ATCG00840	Ribosomal protein L23	-1,78473
AT5G58230	Arabidopsis multicopy suppressor of IRA1	-2,11341

Supplementary Table S5. Proteins related to defense response modulated uniquely in infected WT plants.

Gene ID	Gene name	Log_2 FC
AT3G44300	Nitrilase 2	5.96
AT1G22410	Class-II DAHP synthetase	3.23
AT1G04980	Protein disulfide isomerase 10	3.03
AT3G46280	Protein kinase-related	2.94
AT1G02930	Glutathione transferase	2.59
AT1G17745	3-Phosphoglycerate dehydrogenase	2.46
AT5G52640	HSP90-1	2.24
AT3G19010	2-Oxoglutarate and Fe(II)-dependent oxygenase	1.33
AT2G01490	Phytanoyl-Coa 2-hydroxylase	1.19
AT5G66190	Leaf-type ferredoxin:NADP(H) oxidoreductase	-1.07
AT4G14890	Ferredoxin c 1	-1.023
AT4G21960	Prxr1	-2.01
AT3G60130	Beta glucosidase 16	1.52
AT3G08510	Phospholipase C2	-13.06

SUPPLEMENTARY METHODS

Proteomic analysis

Protein extraction and digestion

Approximately 70 mg of leaf material for each sample was homogenized in 100 µL extraction buffer containing 4% SDS, 5% glycerol, 40 mM Tris-Cl (pH 6.8) and 2x Proteinase Inhibitor Cocktail (Sigma-Aldrich, St Louis, Missouri, USA). Samples were centrifuged at 20,870 × *g* for 15 min at 4°C, and the supernatant was decanted and centrifuged under the same conditions. The protein content was determined using the bicinchoninic acid (BCA) assay (Sigma-Aldrich) with BSA as a standard. The protein extract (200 µg) was then centrifuged at 10,000 × *g* through a cellulose filter column (Microcon 30-kDa Centrifugal Filter Units; Merck Millipore, Billerica, Massachusetts, USA) at 4°C for 5 min using the FASP protocol (Manza *et al.* 2005, Wisniewski *et al.* 2009). The recovered protein was digested with 8 µg trypsin (Promega Corp., Fitchburg, Wisconsin, USA) in 100 mM ammonium bicarbonate and incubated overnight at 37°C in darkness, shaking at 350 rpm. Peptides were collected by centrifugation at 20,870 × *g* for 40 min. Filters were washed with 50 µl 0.5 M NaCl, and centrifuged at 20,870 × *g* for 20 min to collect the remaining peptides. Eluted peptides were acidified with 1% trifluoracetic acid (TFA), desalted using SepPack columns (Tecknokroma, Barcelona, Spain), eluted in 60% acetonitrile containing 0.1% TFA, dried in a Savant SPD131DDA SpeedVac (Thermo Fisher Scientific) and stored at –20°C.

Mass spectrometry

Peptides were re-suspended in 40 µL 5% v/v acetonitrile, 2% v/v TFA and analyzed using a Q Exactive Plus coupled to an Easy nLC1000 HPLC (Thermo Fisher Scientific). Samples were loaded onto an Acclaim PepMap RSLC reversed-phase column (75 µm inner diameter, 25 cm long, Thermo Fisher Scientific) at a flow rate of 0.4 µL/min in 3% v/v acetonitrile, 0.5% v/v acetic acid, and eluted at a flow rate of 0.3 µL/min and an acetonitrile gradient of 3% to 30% v/v over 200 min, 30% to 40% over 20 min, and 40% to 60% over 20 min, followed by a washing step with 90% v/v

acetonitrile for 10 min. Peptide ions were detected in a full scan (*m/z* 200–1600). MS/MS scans were performed for the 10 peptides with the strongest MS signal (AGC target 1e5, isolation width mass-to-charge ratio 3 *m/z*, relative collision energy 30%). Peptides for which MS/MS spectra had been recorded were excluded from further MS/MS scans for 20 s.

<u>Protein quantitation and statistical analysis</u>

Quantitative MS/MS analysis was carried out using Progenesis QI (Nonlinear USA, Durham, North Carolina, USA). Proteins were identified from spectra using Mascot (Matrix Science, Boston, Massachusetts, USA) with the following search parameters: TAIR10 protein annotation, requirement for tryptic ends, one missed cleavage allowed, fixed modification: carbamidomethylation (cysteine), variable modification: oxidation (methionine), peptide mass tolerance = ± 10 ppm, MS/MS tolerance = ± 0.6 Da, allowed peptide charges of +2 and +3. A decoy database search was used to limit false discovery rates (FDRs) to 1% at the protein level. Peptide identifications below rank one or with a Mascot ion score below 25 were excluded. Mascot results were imported into Progenesis QI, quantitative peak area information was extracted and the results exported for data plotting and statistical analysis.

RNA-Seq analysis

<u>Library preparation</u>

The quantity and purity of total RNA samples were assessed by Nanodrop 1000 spectrophotometry and integrity was determined using the RNA 6000 Nano Kit (Agilent Technologies Inc., Santa Clara, California, USA). RNA-Seq libraries were prepared starting from 2.5 µg of total RNA using the TruSeq RNA Sample Prep Kit v2 according to the manufacturer's instructions (Illumina Inc., San Diego, California, USA). The quality of the libraries was checked using the High Sensitivity DNA Kit (Agilent). Libraries were sequenced on an Illumina HiSeq 1000 sequencer using TruSeq SBS Kit v3, and 50 bp paired-end sequences were generated.

Differential gene expression analysis

Bcl conversion and demultiplexing were carried out using CASAVA pipeline v1.8.2 (Illumina). For sequence alignment, a reference sequence was built including the *A. thaliana* TAIR10 reference genome (ftp://ftp.arabidopsis.org/home/tair/) and the sequence of the construct containing the rat (*Rattus norvegicus*) GC (NCBI Gene IDs 497757 and 25202). Reads were aligned using TopHat v2.0.12 with the "b2 very sensitive" parameter. Expression values (fragments per kilobase of exon per million reads mapped, FPKM) were calculated using Cufflinks v2.2.0 with default parameters and Cuffdiff was used to identify differentially expressed genes according to the following criteria: FDR ≤ 5%, log2(FC) ≥ |1| or log2FC ≥ |0.5| as indicated, FPKM > 0.1.
